# Wnt-Dependent Inactivation of the Groucho/TLE Co-repressor by the HECT E3 Ubiquitin Ligase Hyd/UBR5

**DOI:** 10.1016/j.molcel.2017.06.009

**Published:** 2017-07-20

**Authors:** Joshua E. Flack, Juliusz Mieszczanek, Nikola Novcic, Mariann Bienz

**Affiliations:** 1 MRC Laboratory of Molecular Biology, Cambridge Biomedical Campus, Francis Crick Avenue, Cambridge, CB2 0QH, UK

**Keywords:** Wnt/β-catenin signaling, Groucho/TLE repression, HECT E3 ubiquitin ligase, VCP/p97

## Abstract

Extracellular signals are transduced to the cell nucleus by effectors that bind to enhancer complexes to operate transcriptional switches. For example, the Wnt enhanceosome is a multiprotein complex associated with Wnt-responsive enhancers through T cell factors (TCF) and kept silent by Groucho/TLE co-repressors. Wnt-activated β-catenin binds to TCF to overcome this repression, but how it achieves this is unknown. Here, we discover that this process depends on the HECT E3 ubiquitin ligase Hyd/UBR5, which is required for Wnt signal responses in *Drosophila* and human cell lines downstream of activated Armadillo/β-catenin. We identify Groucho/TLE as a functionally relevant substrate, whose ubiquitylation by UBR5 is induced by Wnt signaling and conferred by β-catenin. Inactivation of TLE by UBR5-dependent ubiquitylation also involves VCP/p97, an AAA ATPase regulating the folding of various cellular substrates including ubiquitylated chromatin proteins. Thus, Groucho/TLE ubiquitylation by Hyd/UBR5 is a key prerequisite that enables Armadillo/β-catenin to activate transcription.

## Highlights

• Wnt-dependent inactivation of Groucho/TLE requires ubiquitylation by Hyd/UBR5
• Inactivation of ubiquitylated Groucho/TLE also depends on VCP/p97
• Groucho/TLE ubiquitylation by Hyd/UBR5 enables β-catenin-dependent transcription

## Graphical Abstract

**Figure:**
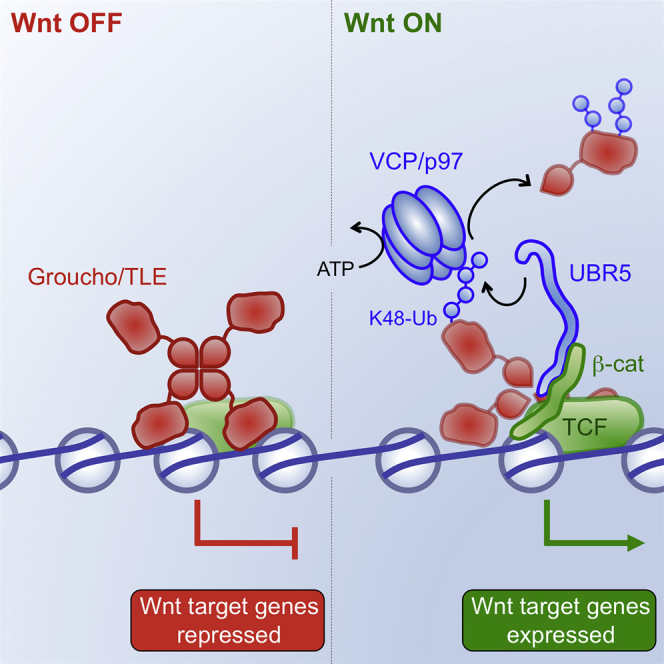

## Teaser

Flack et al. discover that the transcriptional co-repressor Groucho/TLE is disabled during Wnt signaling by ubiquitylation by the HECT E3 ligase Hyd/UBR5. This Hyd/UBR5-dependent inactivation of Groucho/TLE also involves VCP/p97, an AAA ATPase regulating the folding of various cellular substrates.

## Introduction

During the development of animals, cell fates are specified by a handful of highly conserved cell communication pathways that operate context-dependent transcriptional switches (Barolo and Posakony, 2002). An ancient example is the Wnt/β-catenin signaling pathway, highly conserved from the most primitive animals to humans, which controls numerous cell fate decisions during development (Cadigan and Nusse, 1997). This pathway also operates in stem cell compartments of adult tissues, likely explaining why its dysregulation often leads to cancer. Indeed, the great majority of colorectal cancers are initiated by aberrant activation of β-catenin in the intestinal epithelium (Clevers and Nusse, 2012).

The transduction of the Wnt signal from the cell membrane to the nucleus is understood in outline (MacDonald et al., 2009). This crucially depends on the β-catenin effector, which is highly unstable in the absence of a Wnt signal, owing to phosphorylation of specific sites in its N terminus. These phosphorylations are imparted by two kinases (glycogen synthase kinase 3, GSK3, primed by casein kinase 1α, CK1) within a multiprotein complex assembled by Axin and the APC tumor suppressor (“Axin degradasome”; Mendoza-Topaz et al., 2011), which targets β-catenin for ubiquitylation and proteasomal degradation. Wnt signaling inhibits the Axin degradasome, allowing β-catenin to accumulate in the cytoplasm and nucleus. This enables β-catenin to gain access to TCF factors bound to Wnt-responsive enhancers, which are kept silent prior to Wnt signaling by TCF-associated Groucho/TLE co-repressor. Once bound to TCF, β-catenin activates the transcription of downstream target genes, by recruiting a series of transcriptional co-activators via its C terminus, including chromatin remodelers and modifiers (Mosimann et al., 2009).

Groucho/TLE is tethered to enhancers by DNA-binding proteins including HES (Hairy/Enhancer-of-Split), RUNX, and TCF and appears to repress transcription of linked genes primarily by chromatin compaction (Sekiya and Zaret, 2007), although histone deacetylation also plays a role (Jennings and Ish-Horowicz, 2008, Turki-Judeh and Courey, 2012). In the case of TCF, Groucho/TLE-dependent repression is overcome by stabilized (i.e., activated) β-catenin, but how this is achieved is unclear. Initially, it was thought to rely on displacement of Groucho/TLE by β-catenin from TCF by direct competition for binding, but this now seems unlikely since β-catenin and TLE1 can bind simultaneously to TCF (Chodaparambil et al., 2014). Indeed, a recent genome-wide study in *Drosophila* found Groucho to be associated with target genes regardless of their activity, leading the authors to conclude that the repressive activity of Groucho does not depend on its recruitment to targets (Chambers et al., 2017).

Consistent with this, Groucho/TLE is an integral component of a multi-protein transcription complex termed the Wnt enhanceosome, helping to tether this complex to transcriptional enhancers via its association with TCF to earmark them for timely Wnt responses (Fiedler et al., 2015, van Tienen et al., 2017). The Wnt responsiveness of this complex is conferred by Pygopus (Pygo) which binds to its core module Chip/LDB-SSDP (ChiLS) and captures stabilized Armadillo/β-catenin via the Legless/BCL9 adaptor (Kramps et al., 2002, Townsley et al., 2004). In *Drosophila*, Pygo function becomes largely dispensable if Groucho is eliminated by mutation (Mieszczanek et al., 2008), suggesting that Pygo enables Armadillo to access TCF and overcome Groucho-dependent repression. But how TCF-bound Armadillo/β-catenin inactivates the repressive function of Groucho/TLE remains an open question and is likely to require further co-factors.

Here, we report that the HECT E3 ubiquitin ligase Hyperplastic discs (Hyd) and its human ortholog UBR5 (also known as EDD1) are crucial co-factors of Armadillo/β-catenin, enabling it to relieve Groucho/TLE-dependent repression. Loss-of-function and epistasis analyses show that Hyd/UBR5 is required downstream of Armadillo/β-catenin for transcriptional Wnt responses in *Drosophila* wing imaginal discs and human cell lines. Its relevant substrate is Groucho/TLE, whose ubiquitylation by UBR5 is Wnt-inducible and conferred by β-catenin. Our evidence implicates valosin-containing protein (VCP, also known as p97) in the UBR5-dependent inactivation of TLE. We have thus uncovered a mechanism by which Hyd/UBR5 and VCP/p97 co-operate to overcome Groucho/TLE-dependent repression of transcription.

## Results

### *hyd* Is Essential for the Activity of Stabilized Armadillo

**Figure 1:**
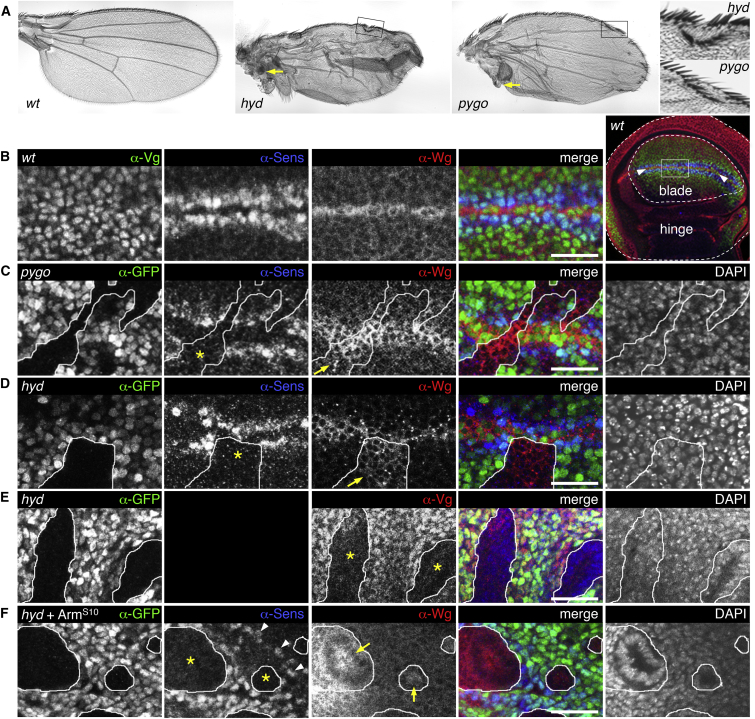
*hyd* Is Required for Wg Responses Downstream of Armadillo (A) Wings with mutant clones (as labeled), showing margin defects (boxed; higher magnification on the right) and overgrowths in the hinge (arrows); WT wing on the left. (B–F) Sections of wing discs from late third-instar larvae, fixed and co-stained with DAPI (blue) and antibodies as indicated above panels (in color, as in merges); (B) WT disc (as boxed in low-magnification view on the right, showing prospective hinge zone surrounding wing blade, delineated by dotted lines, with prospective margin between arrowheads); discs bearing (C) *pygo*^*S123*^ or (D–F) *hyd*^*K7-19*^ mutant clones (marked by absence of GFP, green), (F) also expressing Arm^S10^. Note the lack of Vg and Sens within clones near the margin (asterisks), which also show derepressed Wg (arrows), leading to ectopic Sens in adjacent WT cells (arrowheads). Size bars, 10 μm. See also Figure S1.

Previous work indicated that UBR5 affects Wnt/β-catenin signaling in human cell lines; however in one study, UBR5 seemed to negatively regulate β-catenin, destabilizing it via upregulation of APC (Ohshima et al., 2007), whereas in another, UBR5 positively regulated the stability of β-catenin via assembling on it non-canonical ubiquitin (Ub) conjugates (linked via lysine 11 and 29, K11 and K29; Hay-Koren et al., 2011). To resolve this discrepancy, we examined the consequences of Hyd loss on Wingless (Wg) responses in *Drosophila*, by generating *hyd* mutant clones in wing imaginal discs (note that *hyd* is essential for viability, and for germline development, which precludes analysis of embryonic stages; Mansfield et al., 1994). These *hyd* mutant clones produce wing phenotypes similar to those caused by *pygo* mutant clones, with margin defects accompanied by ectopic margin bristles (Figure 1A).

Next, we monitored Wg target gene expression in wing discs by staining clone-bearing discs with antibodies against Senseless (Sens) and Wg: *wg* is expressed in a stripe along the prospective wing margin where it progressively narrows its own expression by a negative feedback loop (Rulifson et al., 1996) while activating *sens* in neighboring cells (Figure 1B). Accordingly, *sens* expression is eliminated in *pygo* mutant clones near the margin (Parker et al., 2002), while *wg* is derepressed within these clones (Figure 1C). The same is true in *hyd* mutant clones, although their phenotypes are somewhat stronger (Figure 1D). We also examined *vestigial* (*vg*), another Wg target gene expressed in a broad domain straddling the margin (Schweizer et al., 2003), which is downregulated in *pygo* mutant clones in the prospective wing blade (Fiedler et al., 2015), and likewise in *hyd* mutant clones (Figure 1E). In other words, *hyd* mutant clones phenocopy *pygo* mutant clones, causing loss of Wg responses in the wing disc. Hyd, like Pygo, is thus a positive regulator of Wg signaling in this tissue. We also note that the hyperplastic phenotype initially described in *hyd* hypomorphic flies (Mansfield et al., 1994) can be ascribed to SoxF (Figure S1), a repressor of proliferation activated by Wg in various tissues including the prospective wing hinge (Dichtel-Danjoy et al., 2009). In other words, this hyperplastic phenotype further supports the notion of Hyd as a positive regulator of Wg responses in the wing disc. *hyd* mutant germ cells do not develop, and we therefore cannot analyze *hyd* function at earlier developmental stages.

Given the striking similarities between *hyd* and *pygo* mutant phenotypes, we asked whether *hyd* blocks the activity of stabilized Armadillo (called Arm^S10^; Pai et al., 1997), as *pygo* does in fly embryos (Thompson et al., 2002). Indeed, *hyd* mutant clones expressing Arm^S10^ invariably lack Sens (although *sens* is ectopically activated by Arm^S10^ in adjacent wild-type [WT] cells) but exhibit derepressed Wg staining (Figure 1F). Essentially the same is seen in double mutant *hyd axin* clones (Figure S1). We conclude that *hyd* acts downstream of stabilized Armadillo, like Pygo (Kramps et al., 2002, Parker et al., 2002, Thompson et al., 2002). Notably, the levels of Armadillo are normal in *hyd* mutant clones (Figure S1), as in *pygo* mutant clones (Parker et al., 2002, Thompson et al., 2002). This appears to contrast with human cells whose β-catenin seemed stabilized (Ohshima et al., 2007) or destabilized (Hay-Koren et al., 2011) after UBR5 depletion. However, in our hands, siRNA-mediated depletion of UBR5 (with several different siRNAs, including those used by these authors) proved highly unreliable and produced inconsistent effects on β-catenin levels and activity (N.N., unpublished data).

### UBR5 Is Required for Efficient Transcriptional Activity of β-Catenin in Human Cells

**Figure 2:**
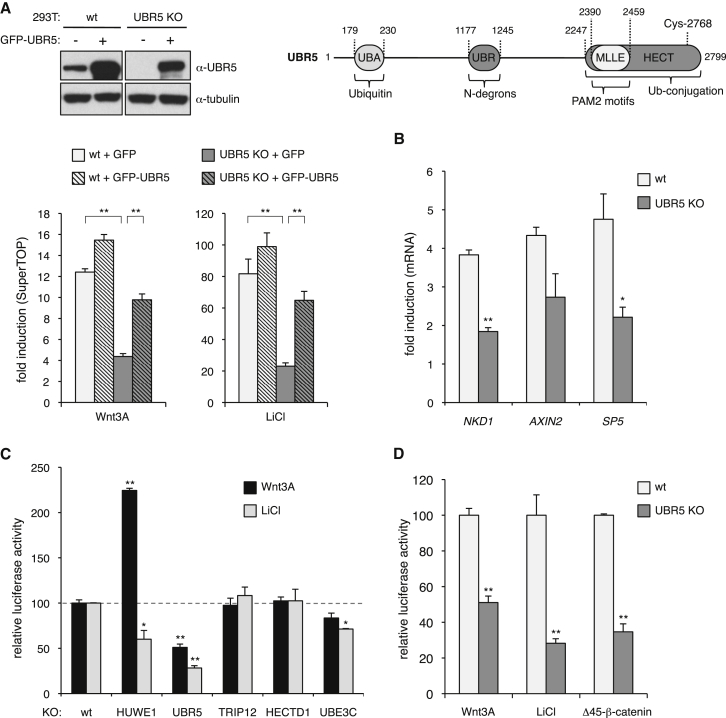
*UBR5* Is Required for β-Catenin-Dependent Transcription in Human Cells (A) Top: cartoon of UBR5 and its domains, with cognate ligands (residue numbers from human UBR5), and western blot probed with α-UBR5, to assess levels of GFP-UBR5 in UBR5 KO cells relative to endogenous UBR5; bottom: SuperTOP assays of UBR5 KO cells or parental HEK293T controls (WT), transfected with GFP-UBR5 or GFP, ±6 hr of Wnt3A or LiCl (as indicated in key); error bars, SEM of >3 independent experiments; ^∗^p < 0.01, ^∗∗^p < 0.001 (in all panels). (B) RT-qPCR assays of endogenous Wnt target genes in WT or UBR5 KO cells treated with LiCl. (C and D) SuperTOP assays as in (A), comparing (C) WT HEK293T and KO lines lacking different HECT E3 ligases, or (D) responses to Wnt3A, LiCl, and overexpressed HA-Δ45-β-catenin in WT and UBR5 KO cells. See also Figure S2, Tables S1, S2, and S3.

Given this unreliability of UBR5 depletion by RNAi, we decided to delete UBR5 in HEK293T cells by CRISPR/Cas9 (Figure S2, Tables S1 and S2) to test Wnt responses in null mutant human cells (UBR5 KO cells). We used a TCF-dependent reporter assay (called SuperTOP; Veeman et al., 2003) to monitor the transcriptional activity of β-catenin. SuperTOP activity is substantially reduced in UBR5 KO cells treated with Wnt3A or LiCl (which stabilizes β-catenin through inhibition of GSK3), and this transcriptional response can be restored toward normal by re-expression of UBR5 (Figure 2A) but not of catalytically dead UBR5 (UBR5-CS, bearing a cysteine-to-serine substitution, C2768S, in its catalytic site), which has a mild dominant-negative effect (Figure S2). An independently isolated KO line behaved the same (Figure S2). Furthermore, endogenous Wnt target genes such as *NKD1*, *AXIN2*, and *SP5* (Hanson et al., 2012, Lustig et al., 2002) are less inducible in LiCl-stimulated UBR5 KO cells compared to their parental controls (Figure 2B; Table S3). By contrast, deletion of other HECT E3 ligases previously linked to Wnt signaling (HUWE1, HECTD1, UBE3C), or to UBR5 itself (TRIP12) (Figure S2), does not reduce the transcriptional Wnt response in HEK293T cells, although HUWE1 deletion causes hypersensitivity to Wnt3A (Figure 2C), as expected, since HUWE1 negatively regulates the upstream Wnt signaling component Dishevelled (de Groot et al., 2014). Thus, UBR5 is unique among these ligases in behaving as a positive regulator of Wnt-induced transcription in human cells.

Given this equivalence between UBR5 and Hyd, we asked whether UBR5 is required for the transcriptional activity of stabilized β-catenin. We thus expressed an unphosphorylatable mutant of β-catenin (Δ45-β-catenin, found in colorectal cancers; Morin et al., 1997), which is hyperactive in stimulating SuperTOP in WT HEK293T cells, but this activity is much reduced in UBR5 KO cells (Figure 2D). UBR5 thus functions downstream of stabilized β-catenin in human cells, like its Hyd counterpart in flies.

### TLE3 Is Ubiquitylated by UBR5 in a Wnt-Dependent Fashion

From these epistasis analyses, we expected the functionally relevant substrate of Hyd/UBR5 to be downstream of Wnt-activated Armadillo/β-catenin. To identify this substrate, we adopted a proteomics approach based on co-immunoprecipitation (coIP) of proteins associated with FLAG-tagged UBR5 in LiCl-stimulated cells, using a catalytically dead version as bait (FLAG-UBR5-CS), to maximize substrate capture. For comparison, we used a mutant version of FLAG-UBR5-CS lacking its MLLE domain (Figure 2A) embedded in the N terminus of its HECT domain (FLAG-UBR5-CS-ΔMLLE), since this domain binds substrates through a PAM2 motif (Kozlov et al., 2010) and regulates binding of PAM2-motif-containing substrates to the HECT domain (Muñoz-Escobar et al., 2015). Analysis of proteins eluted from these baits by mass spectrometry identified a number of proteins consistently associated with UBR5 baits but not with the control. The UBR5-specific hits found in two independent experiments included several known substrates with a PAM2 motif (e.g., BUB1, BUB1β, and ATXN2L), but the only Wnt signaling component was TLE3, whose association with UBR5 seemed independent of MLLE (Figure S3), consistent with its lack of a recognizable PAM2 motif. We used coIP assays in HEK293T cells co-expressing GFP-UBR5-CS and HA-TLE3 to confirm specific association between these two proteins (Figure S3).

**Figure 3:**
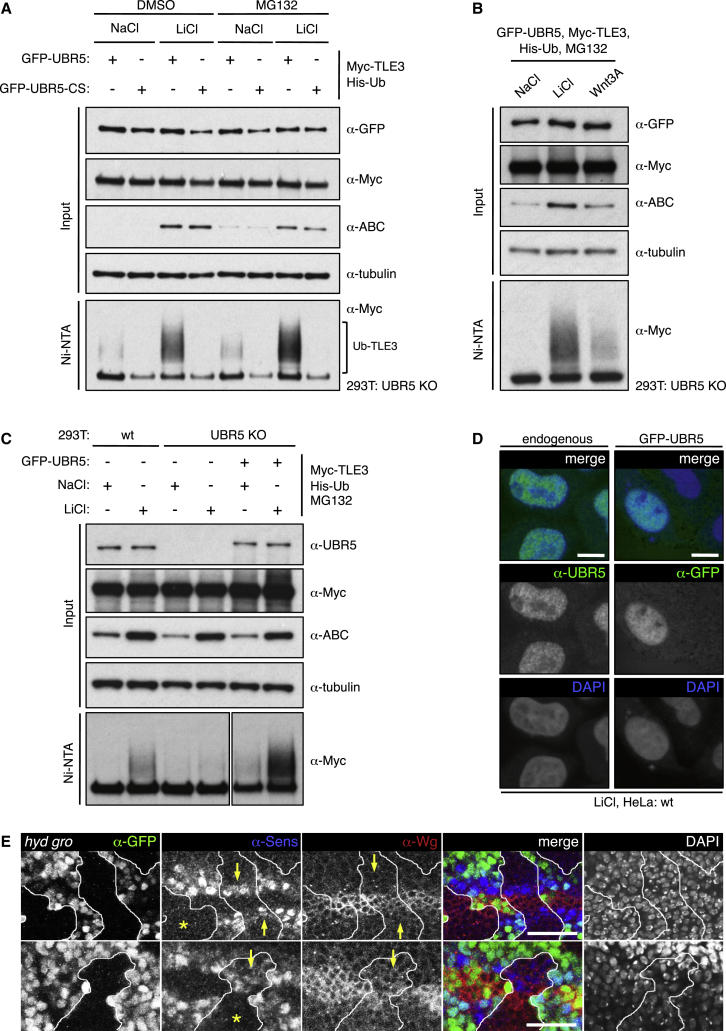
Wnt Signaling Renders Groucho/TLE a Substrate of UBR5 (A) Assays for Ub-TLE3; shown are western blots of UBR5 KO cell lysates, after co-expression of proteins and treatments as indicated above, and affinity purification with Ni-NTA, probed with antibodies as indicated on the right (ABC, active β-catenin, confirming Wnt pathway activation), to reveal Ub-TLE3 (bracketed). (B and C) Assays for Ub-TLE3 as in (A). (D) Confocal sections through HeLa cells ± overexpressed FLAG-UBR5, co-stained with DAPI (blue) and antibodies as labeled (green). (E) *hyd*^*K7-19*^*gro*^*MB36*^ double mutant clones as in Figure 1 (representative examples from two different larvae), with Sens expression and Wg repression (arrows) restored within clones by Groucho loss (compare to *hyd*^*K7-19*^ single mutant clones, Figure 1D); asterisks, examples of clones without restored Sens. Size bars, 10 μm. See also Figures S1 and S3.

To test whether TLE is a substrate of UBR5, we conducted ubiquitylation assays in UBR5 KO cells, co-expressing Myc-TLE3 and GFP-UBR5 (or GFP-UBR5-CS as control) with His-Ub and monitoring polyubiquitylated TLE3 (Ub-TLE3) after affinity purification with Ni-NTA resin. Indeed, GFP-UBR5 but not GFP-UBR5-CS generates Ub-TLE3; notably, this activity is only detectable in LiCl-, but not in mock-, treated cells (Figure 3A). Similarly, exposure of cells to Wnt3A induces UBR5-dependent ubiquitylation of Myc-TLE3 (Figure 3B), while endogenous TLE is also ubiquitylated in LiCl-treated cells in a UBR5-dependent fashion (Figure S3). We also detect substantial levels of LiCl-dependent Ub-TLE3 in WT cells, seemingly conferred by endogenous UBR5 (whose levels are similar to those of GFP-UBR5 re-expressed in UBR5 KO cells; Figure 3C). This striking Wnt-induced ligase activity of UBR5 toward TLE3 indicates that TLE3 is a physiological substrate of UBR5 in cells with an active Wnt pathway. Consistent with this, both endogenous and re-expressed GFP-UBR5 are confined to the nucleus (Hay-Koren et al., 2011) (Figure 3D), and so most physiological substrates of UBR5 are expected to be nuclear proteins.

An important corollary of our findings is that Groucho/TLE is inactivated during Wnt signaling by Hyd/UBR5-dependent ubiquitylation. If so, genetic inactivation of Groucho/TLE should restore Wnt responses in cells lacking Hyd/UBR5. We tested this, by examining Wg responses in *hyd gro* double mutant wing disc clones. Indeed, we observe partial restoration of Sens staining in these double mutant clones near the wing margin (in 22/42 clones scored), but these clones with restored Sens staining do not exhibit Wg derepression (Figure 3E), in contrast to *hyd* mutant clones, which invariably show Wg derepression but never any Sens expression (Figure 1D), or to *gro* mutant clones, which invariably show derepressed Sens in Wg signaling territories (Mieszczanek et al., 2008). In other words, these two different Wg responses that are lost in *hyd* mutant clones are partially (Sens expression) or fully (Wg repression) restored in the double mutant clones. In addition, the SoxF-dependent overgrowth seen in *hyd* mutant clones in the prospective hinge region is fully suppressed in *hyd gro* double mutant clones (Figure S1). Thus, Hyd is dispensable to a large extent for Wg responses in the absence of Groucho, implying that Groucho is a functionally relevant substrate of Hyd in Wg-stimulated cells.

### Stabilized β-Catenin Promotes UBR5-Dependent Ubiquitylation of TLE3

**Figure 4:**
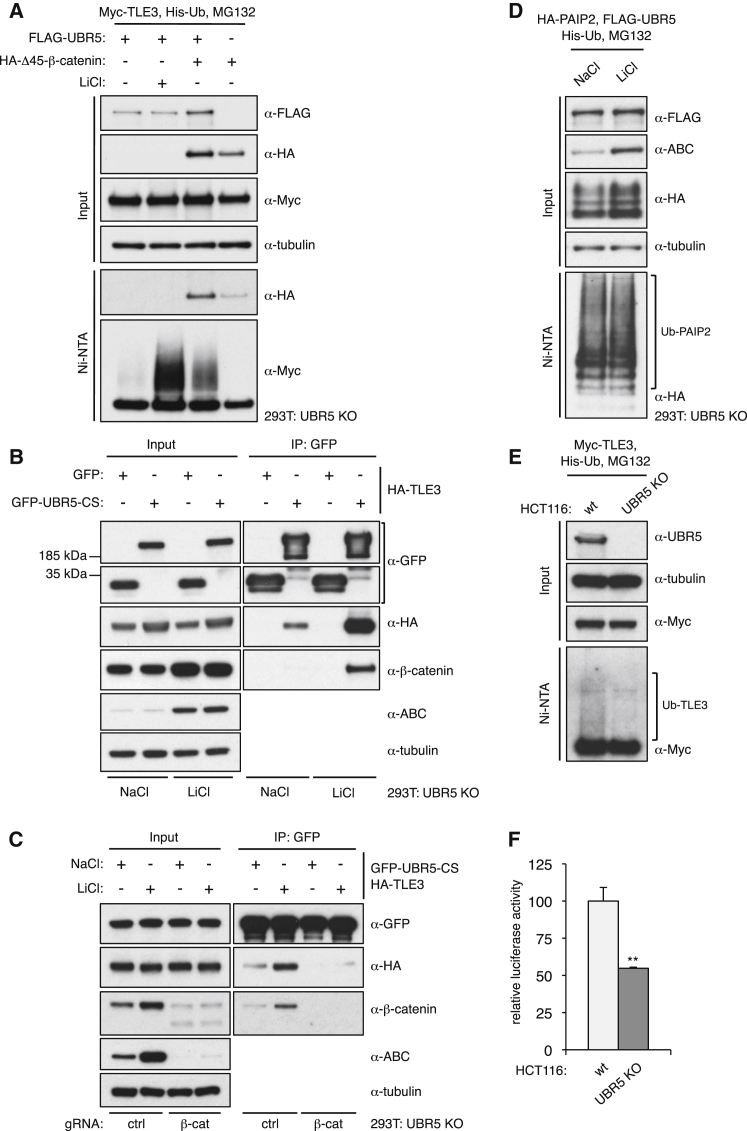
Stabilized β-Catenin Promotes UBR5-Dependent Ub-TLE3 (A) Assays for Ub-TLE3 as in Figure 3A. (B and C) CoIP assays; shown are western blots of UBR5 KO cell lysates, after co-expression of proteins, treatments, and immunoprecipitation (IP) as indicated above and below panels, probed with antibodies as indicated on the right. (D) Assays for Ub-PAIP2, as in (A). (E and F) UBR5 KO HCT116 cells or parental controls (WT), assayed for (E) *in vivo* TLE3-Ub (as in A or F) SuperTOP (as in Figure 2D); error bars, SEM; ^∗∗^p < 0.001.

Given that UBR5 acts below β-catenin, which accumulates during Wnt signaling (Figure 3B), we asked whether stabilized β-catenin by itself could induce UBR5-dependent Ub-TLE3. We expressed Δ45-β-catenin in UBR5 KO cells, with or without resupplied GFP-UBR5, and monitored Ub-TLE3. Indeed, UBR5-dependent Ub-TLE3 is nearly as highly induced by expression of Δ45-β-catenin as by LiCl treatment (Figure 4A). Furthermore, endogenous β-catenin coIPs with GFP-UBR5-CS, but this association is only detectable in LiCl-stimulated cells (Figure 4B). Crucially, the association between GFP-UBR5-CS and HA-TLE3 is strongly increased by LiCl stimulation (Figure 4B) but is barely detectable in β-catenin-depleted cells (Figure 4C). Taken together, these results support the notion that stabilized β-catenin binds to UBR5 and apposes it to TLE3, thereby directing its ubiquitin ligase activity toward TLE3. Notably, this appears to occur within the Wnt enhanceosome, given that FLAG-UBR5-CS coIPs with co-expressed GFP-TCF4 and GFP-PYGO1, regardless of Wnt stimulation (Figure S3). Indeed, in an independent study based on proximity labeling, we detected constitutive association of endogenous UBR5 with the Wnt enhanceosome, near PYGO (van Tienen et al., 2017).

We also considered the possibility that UBR5 may be autoinhibited, like other HECT E3 ligases (see Discussion), and that its disinhibition required β-catenin. However, UBR5 ubiquitylates other substrates in the absence of Wnt pathway activity (Shearer et al., 2015) including PAIP2 (Yoshida et al., 2006). We confirmed that HA-PAIP2 is ubiquitylated efficiently by co-expressed FLAG-UBR5, regardless of Wnt signaling (Figure 4D). Thus, UBR5 is intrinsically active and does not require disinhibition by β-catenin or other factors.

Given that stabilized β-catenin promotes the E3 ligase activity of UBR5 toward TLE3, we asked whether we could detect UBR5-dependent Ub-TLE3 in the colorectal cancer cell line HCT116, whose Wnt pathway activity is elevated due to a Δ45 mutation in one of its β-catenin alleles (Morin et al., 1997). We thus used the CRISPR/Cas9 system to delete UBR5 in these cells and examined their Ub-TLE3. Indeed, Ub-TLE3 is detectable in the parental HCT116 line but not in the UBR5 KO derivatives (Figure 4E). Furthermore, the β-catenin-dependent transcription is significantly reduced in the KO cells compared to their parental controls (Figure 4F). This corroborates the results from our epistasis analysis, underscoring the notion that stabilized β-catenin is sufficient to direct UBR5 activity toward TLE3 to inhibit its repressive function.

### UBR5 Ubiquitylates the Ligand-Binding WD40 Domain of TLE3

**Figure 5:**
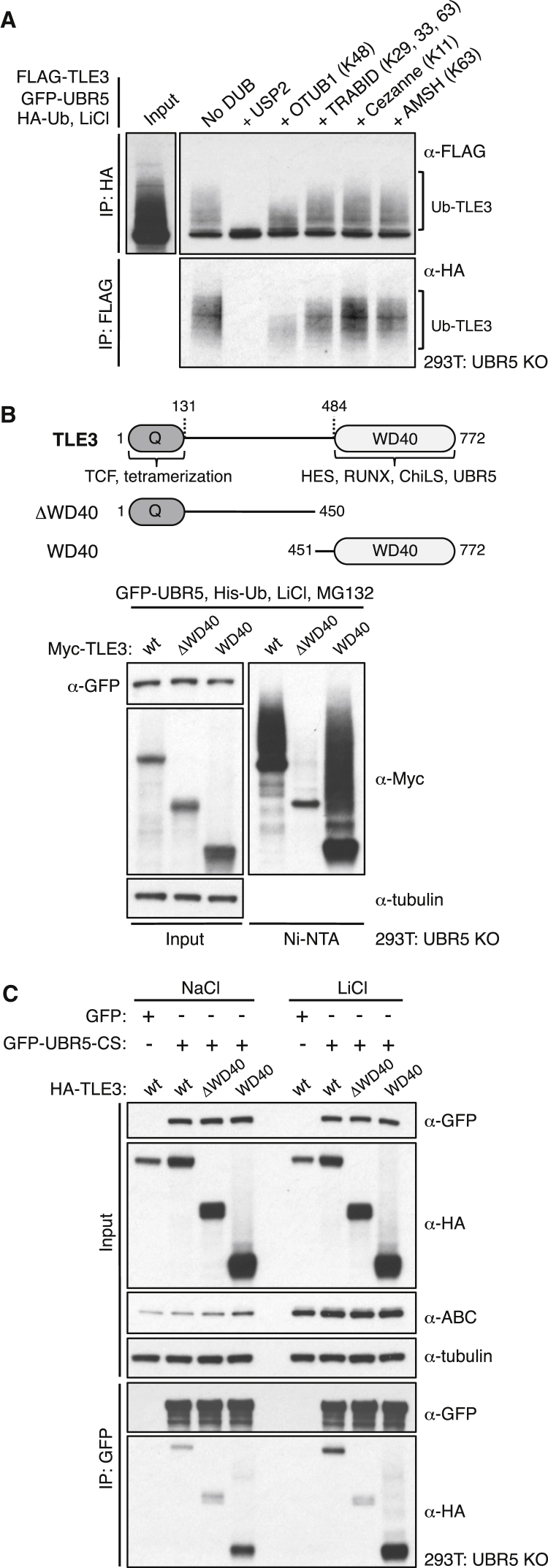
UBR5 Ubiquitylates the WD40 Domain of TLE3 (A) UbiCREST assays of Ub-TLE3; shown are western blots of UBR5 KO lysates after co-expression of proteins, LiCl induction and IP as indicated, followed by *in vitro* treatment of IPs with linkage-specific DUBs (specificity in brackets), or USP2 (unspecific control). (B and C) UBR5 KO cells, assayed for (B) Ub-TLE3 (as in Figure 3A) or (C) coIP of HA-TLE3 and truncations (see cartoon, for residue numbers, domains, and cognate ligands) with GFP-UBR5-CS (as in Figure 4B). See also Figures S4 and S5.

Previous evidence based on cells expressing ubiquitin mutants with only a single lysine available for conjugation (K-only Ub) suggested that UBR5 modifies β-catenin with “non-canonical” ubiquitin conjugates (linked at K29 and K11; Hay-Koren et al., 2011) in cells. However, the UBR5 construct used by these authors bore a His tag at its C terminus, and we confirmed that this inactivated its E3 ligase activity (N.N., unpublished data), as might be expected (Salvat et al., 2004). Removing this tag revealed the activity of UBR5 toward TLE3 and PAIP2 (see above), yet ubiquitylation of β-catenin was not detectable in the same lysates (Figure 4A). We thus also re-examined the linkage specificity of UBR5, using an *in vitro* Ub assay supplemented with K-only Ub mutants (bearing arginine-to-lysine substitutions at all but one lysine) and bacterially expressed HECT domain of UBR5 (UBR5_2217-2799_). UBR5_2217-2799_ auto-ubiquitylates when supplied with WT Ub, and with K48-only Ub, but not with any of the other K-only Ub mutants (Figure S4). Consistent with this, analysis of affinity-purified FLAG-TLE3 by mass spectrometry (see below) revealed di-Ub peptides exclusively derived from K48-linked Ub in addition to unlinked Ub peptides (while parallel affinity purifications of Dishevelled revealed association with K11-linked Ub; Mund et al., 2015). We used UbiCREST assays (Hospenthal et al., 2015), cleaving Ub-TLE3 with deubiquitylases (DUBs) specific for K11-, K29-, K33-, K48-, and K63-linked Ub, to show that only the K48-specific enzyme is active toward Ub-TLE3 (Figure 5A), confirming that UBR5 generates K48-Ub conjugates.

A likely implication is that UBR5 substrates are targeted for proteasomal degradation by their K48-Ub chains. Indeed, the levels of Ub-TLE3 are elevated after proteasome inhibition (Figure 3A). However, cycloheximide chase experiments did not reveal any differences in the steady-state levels of TLE3 between LiCl-stimulated WT and UBR5 KO cells (Figure S4). Likewise, the levels of Gro staining are unaltered within *hyd* mutant clones compared to their neighboring WT cells (J.M., unpublished data). While this suggests that UBR5 does not primarily target TLE for proteasomal degradation, it is possible that these assays monitoring bulk Groucho/TLE are not sensitive enough to detect UBR5-dependent destabilization of the TCF-associated pool of TLE. Furthermore, the rate of Ub-TLE turnover may be too slow to be detected in these short-term assays.

Next, we mapped the domain of TLE3 ubiquitylated by UBR5, by generating various TLE3 truncations. This revealed that the C-terminal WD40 domain of TLE3 is both necessary and sufficient for UBR5-dependent ubiquitylation in LiCl-stimulated cells (Figure 5B). As expected, this domain coIPs with GFP-UBR5-CS, and this interaction is enhanced by LiCl (Figure 5C). Thus, TLE3 interacts with UBR5 via its WD40 domain, which becomes ubiquitylated as a result. We also examined the *in vivo* ubiquitylation of individual K-only WD40 mutants, which revealed that most of these mutants can be ubiquitylated efficiently, with K720-only being one of the strongest ubiquitin acceptors (Figure S5). Notably, K720 is solvent-exposed within the pore of the WD40 propeller domain and is crucial for its binding to a short C-terminal motif in HES and RUNX proteins (Jennings et al., 2006). Evidently, this and other lysines on either propeller surface can serve as Ub attachment sites for UBR5.

The WD40 domain is also required for TLE’s association with ChiLS (Fiedler et al., 2015), and for its binding to nucleosomal arrays (Sekiya and Zaret, 2007). We thus asked whether the ubiquitylation of WD40 would block its binding to these ligands. However, Ub-WD40 coIPs efficiently with GFP-HES1 or GFP-ChiLS, and Ub-TLE3 is also pulled down by biotinylated histone H2A, H3, and H4 tails (see Chodaparambil et al., 2014) comparably to unmodified TLE3 (Figure S5). Thus, the ubiquitylation of WD40 does not block its ligand binding. We note however that these coIP assays have technical limitations, and we suspect would not be able to detect subtle attenuations of ligand binding that could be crucial in cells.

### VCP/p97 Is Required for UBR5-Dependent Inactivation of Ub-TLE

During our attempts to identify Ub attachment sites within the WD40 domain by mass spectrometry, we discovered peptides derived from VCP/p97 in the TLE3 immunoprecipitate. VCP/p97 is an AAA ATPase that has been implicated in the segregation of polyubiquitylated proteins from chromatin in various systems (Dantuma et al., 2014, Xia et al., 2016). We thus wondered whether the inactivation of TLE by UBR5-dependent ubiquitylation might involve VCP/p97.

**Figure 6:**
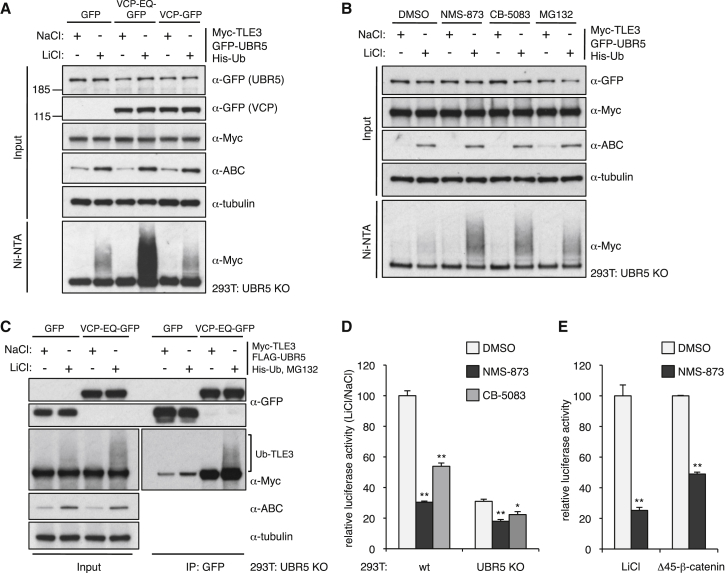
VCP/p97 Promotes Wnt Responses by Removing UBR5-Dependent Ub-TLE3 (A and B) Assays for Ub-TLE3 as in Figure 3A, after (A) co-expression of catalytically dead VCP/p97 (VCP-EQ-GFP) or (B) treatment with VCP/p97 inhibitors (NMS-873 or CB-5083). (C) CoIP assays (as in Figure 4B), showing constitutive association of dominant-negative VCP/p97 (VCP-EQ-GFP) with TLE3. (D and E) SuperTOP assays (as in Figure 2D); error bars, SEM; ^∗^p < 0.01, ^∗∗^p < 0.001. See also Figure S6.

To test this, we co-expressed a widely used catalytically dead version of this ATPase (bearing a glutamate-to-glutamine substitution, E578Q, in its D2 catalytic pocket; VCP-EQ-GFP) in UBR5 KO cells and examined Ub-TLE3 after re-supply of UBR5 and LiCl stimulation. We thus found the levels of UBR5-dependent Ub-TLE3 to be strongly increased by VCP-EQ-GFP (Figure 6A). The same is true if the transfected cells are treated with NMS-873, a highly specific allosteric inhibitor of VCP/p97 (Magnaghi et al., 2013), or with a distinct VCP/p97 inhibitor called CB-5083, which acts ATP-competitively through its D2 catalytic pocket (Zhou et al., 2015). In fact, these inhibitors are more effective in elevating the Ub-TLE3 levels than proteasome inhibition by MG132 (Figure 6B), which we used in most of our experiments. Furthermore, Ub-TLE3 also coIPs with catalytically dead VCP/p97 (Figure 6C). Taken together, these results suggest a functional link between VCP/p97 and Ub-TLE3.

Finally, we asked whether these interactions between VCP/p97 and TLE3 are functionally relevant for Wnt signaling, by monitoring β-catenin-dependent transcription in WT or UBR5 KO cells with or without VCP-EQ-GFP, or after VCP/p97 inhibitor treatment. Indeed, the LiCl-induced SuperTOP activity is severely reduced in WT HEK293T cells under both conditions of VCP/p97 inhibition, but barely affected in UBR5 KO cells (Figure 6D). If this activity is restored in the KO cells by UBR5 re-expression, this also restores sensitivity to VCP/p97 inhibition (Figure S6). Notably, SuperTOP is also sensitive to VCP/p97 inhibition by NMS-873 if this reporter is induced by Δ45-β-catenin in WT HEK293T cells (Figure 6E), supporting the notion that the functionally relevant target of VCP/p97 is in the downstream (nuclear) part of the Wnt signaling cascade. Finally, NMS-873 also attenuates the LiCl inducibility of endogenous Wnt target genes in WT cells, but barely in UBR5 KO cells (Figure S6). Thus, our results implicate VCP/p97 in the Wnt-dependent inactivation of Ub-TLE3.

## Discussion

**Figure 7:**
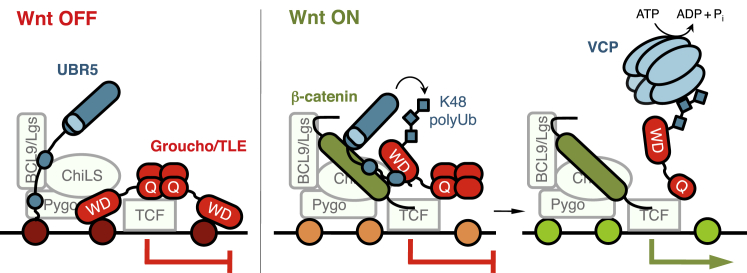
Model of Groucho/TLE Inactivation by Hyd/UBR5 and VCP/p97 Left: prior to Wnt signaling, the Groucho/TLE tetramer represses transcription of TCF target genes by chromatin compaction (nucleosomes in crimson). Right: upon Wnt signaling, stabilized β-catenin docks the Wnt enhanceosome and induces a conformational change (van Tienen et al., 2017) that results in the apposition of UBR5 to TCF-bound Groucho/TLE, enabling UBR5 to attach K48-Ub to Groucho/TLE. This renders it a substrate for VCP/p97-dependent unfolding (possibly facilitated by the Ub-dependent loosening of Groucho/TLE’s contacts with nucleosomes, in orange), which destabilizes the Groucho/TLE tetramer, and thus relieves chromatin compaction (nucleosomes in light green), allowing transcriptional activation.

An essential step enabling Wnt-dependent transcription is the conversion of the Wnt enhanceosome from silent to active. This involves the binding of the Wnt effector β-catenin to TCF, which releases the transcriptional silence imposed on the linked genes by TCF-bound Groucho/TLE. We have discovered a crucial role of Hyd/UBR5 in this process, and our evidence suggests that β-catenin directs the activity of this HECT ubiquitin ligase toward Groucho/TLE, to block its repressive activity (Figure 7). Our evidence also implicates VCP/p97 in this UBR5-dependent inactivation of Groucho/TLE during Wnt signaling.

### Groucho/TLE Is a Physiological Substrate of Hyd/UBR5 in Wnt-Stimulated Cells

By generating UBR5 null mutant cell lines, we were able to resolve previous inconsistencies regarding the effects of UBR5 depletion on Wnt/β-catenin responses in human cell lines (Hay-Koren et al., 2011, Ohshima et al., 2007). Our UBR5 KO cell lines consistently showed reduced Wnt responses, but no changes in β-catenin levels. This parallels our results from *hyd* mutant clones in flies, providing unequivocal evidence for Hyd/UBR5 as a positive regulator of Wnt signaling in fly and human cells.

Three strands of evidence implicate Groucho/TLE as a physiologically relevant substrate of Hyd/UBR5 during Wnt signaling. First, our epistasis analysis revealed that Hyd/UBR5 acts below Armadillo/β-catenin, and thus likely targets a substrate in the nucleus, consistent with its nuclear localization. Second, the activity of UBR5 in ubiquitylating Groucho/TLE is triggered by Wnt/β-catenin signaling. Third, in *Drosophila* wing discs, *hyd* is largely dispensable in the absence of Groucho (as revealed by *hyd gro* double mutant clones), which provides powerful evidence that Hyd acts by antagonizing Groucho.

### The Wnt-Induced Ligase Activity of UBR5 toward TLE Is Conferred by β-Catenin

We considered two possible mechanisms by which β-catenin might activate UBR5 toward TLE3 during Wnt signaling. Either, β-catenin might disinhibit UBR5 if this enzyme were normally autoinhibited, like the NEDD4 family HECT ligases (e.g., Mari et al., 2014, Mund and Pelham, 2009). Indeed, one of these ligases (WWP2) is disinhibited by Dishevelled, which, upon polymerization, engages in multivalent interactions with WWP2 to release its cognate binding sites from autoinhibitory contacts (Mund et al., 2015). However, the strong activity of UBR5 toward PAIP2 in the absence of Wnt signaling (Figure 4D) argues against this mechanism. We thus favor an alternative mechanism, namely that β-catenin apposes enzyme and substrate, e.g., via triggering a conformational change of the Wnt enhanceosome that results in proximity between UBR5 and Groucho/TLE (Figure 7). Support for this mechanism comes from previous proximity labeling experiments that revealed a β-catenin-dependent rearrangement of some of the components within the Wnt enhanceosome (van Tienen et al., 2017), and from our coIP assays showing that β-catenin promotes the association between UBR5 and TLE3 (Figures 4B and 4C).

### Inactivation of Groucho/TLE by UBR-Dependent Ubiquitylation

How does UBR5-dependent ubiquitylation of Groucho/TLE inactivate its co-repressor function? The most obvious mechanism involves proteasomal turnover of Ub-TLE, given the specificity of UBR5 in generating K48-linked Ub chains, which are efficient proteasomal targeting signals. In support of this, the levels of UBR5-dependent Ub-TLE3 are elevated after proteasome inhibition. However, our negative results from the cycloheximide chase experiments argue against rapid proteosomal degradation being the primary mechanism underlying the UBR5-dependent inactivation of Groucho/TLE.

We also considered that the ubiquitylation of the WD40 domain might interfere with its binding to cognate ligands, and thus weaken the association of Groucho/TLE with the Wnt enhanceosome. However, this does not seem to be the case since Ub-TLE3 appears to bind to its ligands as efficiently as unmodified TLE, including a K-only mutant which can only be ubiquitylated at K720, a WD40 pore residue that is crucial for ligand binding (Jennings et al., 2006) and co-repression (Komachi and Johnson, 1997). Evidently, the extended C terminus through which ubiquitin is attached to K720 is flexible enough to allow simultaneous ligand binding. However, for technical reasons, we have been unable to test the binding of Ub-TLE to the key ligand through which Groucho/TLE exerts its repressive function—namely the nucleosomes to which Groucho/TLE binds via both its structured domains, to promote chromatin compaction (Sekiya and Zaret, 2007). Nevertheless, we consider it plausible that the attachment of multiple ubiquitin chains to the WD40 domain (as indicated by our experiments) would loosen up the binding of Groucho/TLE to nucleosomes, and thus attenuate its ability to compact chromatin.

Our evidence based on dominant-negative VCP/p97 and two distinct VCP/p97 inhibitors (Figure 6) implicates this ATPase in the Wnt-dependent inactivation of Ub-TLE. Intriguingly, a recent proteomic screen for NMS-873-induced VCP/p97-associated proteins identified TLE1 and TLE3 as the only Wnt signaling components, along with VCP/p97 adaptors and other putative substrates (Xue et al., 2016), consistent with our notion of Groucho/TLE as a substrate of this ATPase. VCP/p97 regulates the folding of ubiquitylated proteins, to promote their segregation from large structures, such as endomembranes, and also from large protein complexes, including DNA repair and chromatin complexes (Dantuma et al., 2014, Xia et al., 2016). It is therefore conceivable that VCP/p97 unfolds Groucho/TLE upon its ubiquitylation, especially if this modification loosened the interaction of Groucho/TLE with nucleosomes. Whatever the case, unfolding of the Groucho/TLE tetramer by VCP/p97 is likely to destabilize it, which would disable its repressive function (Song et al., 2004, Chodaparambil et al., 2014). This is consistent with a recent proposal that the relief of Groucho-dependent repression is based on kinetic destabilization of the Groucho complex (Chambers et al., 2017), which may be facilitated by its ubiquitylation and unfolding by VCP/p97.

### Differences between UBR5 and XIAP

One other E3 ligase has been shown to ubiquitylate TLE3, namely the RING ligase XIAP, which constitutively monoubiquitylates the Q domain of TLE3, apparently stimulating Wnt-dependent transcription by blocking its binding to TCF4 (Hanson et al., 2012). This contrasts with the Wnt-induced activity of UBR5 toward TLE3 revealed by our study. Evidently, the two ligases act distinctly, and also independently, given that the UBR5-dependent polyubiquitylation of TLE3 is normal in XIAP KO cells (Figure S7). However, we also noted that the reduction of Wnt-dependent transcription in the XIAP KO cells was modest at best, compared to the substantial reduction in UBR5 KO cells (Figure 2). Either XIAP plays a lesser role in promoting transcriptional Wnt responses or a compensating E3 ligase was upregulated during the process of establishing XIAP KO cells. We note that the XIAP KO mice are viable, and without any overt mutant phenotypes (Harlin et al., 2001), and that the *Drosophila* XIAP mutants do not show *wg*-like phenotypes (e.g., Robbins et al., 2014), in contrast to the *hyd* mutant clones that phenocopy strong *wg*-like mutant phenotypes (Figure 1). All in all, it appears that UBR5 has a more profound role than XIAP in enabling transcriptional Wnt responses.

### Other Functions of UBR5

Inactivation of Groucho/TLE by UBR5 and VCP/p97 (Figure 7) could also underlie other signaling-dependent gene switches that involve Groucho/TLE-dependent repression, e.g., Notch signaling, which depends on binding of Groucho/TLE to HES repressors (Jennings and Ish-Horowicz, 2008, Turki-Judeh and Courey, 2012). Indeed, recent genetic screens in *C. elegans* have identified the UBR5 ortholog *sog-1* as a negative regulator of Notch signaling during nematode development (Safdar et al., 2016). Although it is conceivable that *hyd* also affects Notch responses in flies, we found that the derepression of the Notch target gene *wg* in *hyd* mutant wing disc clones is not sensitive to blockade by dominant-negative Mastermind (Figure S1), which argues against a role of Hyd in Notch-dependent transcription in this tissue. We also note that *Ubr5* has been linked to defective Hedgehog signaling in mice (Kinsella et al., 2016), following an earlier lead of Groucho as a putative Hyd target in the context of Hedgehog signaling (Lee et al., 2002), although these links between Hyd/Ubr5 and Hedgehog signaling appear to be indirect.

However, UBR5 clearly also modifies substrates other than Groucho/TLE, including proteins with PAM2 motifs that are recognized by its MLLE domain (Kozlov et al., 2010), e.g., PAIP2 involved in translational control (Muñoz-Escobar et al., 2015). Furthermore, via its UBR domain (Figure 2A), UBR5 may recognize substrates of the N-end rule pathway (Sriram et al., 2011), though few of these have been identified to date. Given the nuclear location of UBR5, it seems highly likely that most of its physiologically relevant substrates are nuclear proteins, e.g., the RING E3 ligase RNF168, which is ubiquitylated and destabilized by UBR5 during the DNA damage response (Gudjonsson et al., 2012).

### Implications for Cancer

UBR5 has been heavily implicated in cancer, although it is somewhat unclear whether it promotes or antagonizes tumor progression, which may depend on context (Shearer et al., 2015). However, *UBR5* amplification is the predominant genetic alteration in many types of cancers (far more prevalent than loss-of-function *UBR5* mutations), and amplified UBR5 correlates with poor outcomes in breast cancer (Shearer et al., 2015). This implies a tumor-promoting role of UBR5, consistent with its role in relieving Groucho/TLE-dependent repression of Wnt responses. It will be interesting to test whether UBR5 loss-of-function inhibits β-catenin-dependent tumorigenesis, e.g., in the intestine. This might be expected, given our results from the colorectal cancer cell line HCT116 whose β-catenin-dependent transcription is attenuated by UBR5 KO (Figures 4D and 4E) and whose proliferation is slowed down by VCP/p97 inhibition (Magnaghi et al., 2013). If this were to apply generally to other colorectal cancer lines, this would indicate the potential of UBR5 and VCP/p97 as new enzymatic targets for therapeutic intervention in colorectal and other β-catenin-dependent cancers. It could widen the application of CB-5083, an orally bioavailable VCP/p97 inhibitor currently in phase 1 clinical trials (Zhou et al., 2015).

## STAR★Methods

### Key Resources Table

**Table:** 

REAGENT or RESOURCE	SOURCE	IDENTIFIER
**Antibodies**		

α-UBR5	Abcam	Cat#ab70311; RRID: AB_2210186
α-GFP (rabbit)	Sigma	Cat#G1544; RRID: AB_439690
α-GFP (mouse)	Sigma	Cat#G6539; RRID: AB_259941
α-Flag (mouse)	Sigma	Cat#F1804; RRID: AB_262044
α-Flag (rabbit)	Sigma	Cat#F7425; RRID: AB_439687
α-HA (rat)	Sigma	Cat#3F10
α-HA (rabbit)	Abcam	Cat#ab9110; RRID: AB_307019
α-Myc	Santa Cruz Biotechnology	Cat#sc-789; RRID: AB_631274
α-active β-catenin (ABC)	Cell Signaling Technologies	Cat#8814S; RRID: AB_11127203
α-β-catenin	BD Transduction Laboratories	Cat#610153
α-XIAP	BD Transduction Laboratories	Cat#610763; RRID: AB_398086
α-HUWE1	Abcam	Cat#ab70161; RRID: AB_1209511
α-TRIP12	Abcam	Cat#ab86220; RRID: AB_1925533
α-HECTD1	Abcam	Cat#ab101992; RRID: AB_10711075
α-UBE3C	Abcam	Cat#ab101512; RRID: AB_10711205
α-β-tubulin	Sigma	Cat#T4026; RRID: AB_477577
α-TLE1-4	Santa Cruz Biotechnology	Cat#sc-13373; RRID: AB_2203721
α-GST	Abcam	Cat#ab19256; RRID: AB_444809
HRP conjugated Goat α-Mouse	Santa Cruz Biotechnology	Cat#sc-2005; RRID: AB_631736
HRP conjugated Goat α-Rabbit	Santa Cruz Biotechnology	Cat#sc-2301; RRID: AB_650500
HRP conjugated Goat α-Rat	Santa Cruz Biotechnology	Cat#sc-2032; RRID: AB_631755
HRP conjugated Donkey?α-Goat	R&D Systems	Cat#HAF109; RRID: AB_357236
Alexa Fluor 488 conjugated Goat α-Rabbit	Life Technologies	Cat#A11008
Alexa Fluor 488 conjugated Goat α-Mouse	Life Technologies	Cat#A11029
Alexa Fluor 546 conjugated Goat α-Mouse	Life Technologies	Cat#A11003
Alexa Fluor 647 conjugated Goat α-Guinea pig	Invitrogen	Cat#A21450
α-Senseless	Prof. Hugo J. Bellen	N/A
α-Vestigial	Prof. Sean B. Carroll	N/A
α-Wingless	DSHB	Cat#4D4; RRID: AB_528512
α-Armadillo	DSHB	Cat#N27A1; RRID: AB_528089

**Chemicals, Peptides, and Recombinant Proteins**		

Ni-NTA Agarose	QIAGEN	Cat#30210
α-FLAG M2 Affinity Gel	Sigma	Cat#A2220
EZview Red α-HA Affinity Gel	Sigma	Cat#E6779
GFP-trap_A	Chromotek	Cat#gta-20
Glutathione Sepharose 4b	GE Healthcare	Cat#17075601
Dynabeads MyOne Streptavidin C1	Invitrogen	Cat#65001
Lipofectamine2000	Invitrogen	Cat#11668019
Polyethylenimine, linear, MW25000	Polysciences	Cat#23966
EDTA-free Protease Inhibitor Cocktail	Roche	Cat#04693159001
MG132	Sigma	Cat#C2211
NMS-873	Cayman Chemical co.	Cat#1418013-75-8
CB-5083	Cayman Chemical co.	Cat#1542705-92-9
Cycloheximide	Sigma	Cat#C104450
Puromycin dihydrochloride	Sigma	Cat#P8833
3xFLAG-Peptide	Sigma	Cat#F4799
L-Glutathione reduced	Sigma	Cat#G4251
VectaShield with DAPI	Vector Laboratories	Cat#H-1200
Ubiquitin-activating enzyme E1 (UBE1A)	Boston Biochem	Cat#E-305
Ubiquitin-conjugating enzyme E2 (UBE2L3)	Boston Biochem	Cat#E2-640
Ubiquitin	Boston Biochem	Cat#U-100
Methyl-ubiquitin	Boston Biochem	Cat#U-501
K6-only ubiquitin	Boston Biochem	Cat#UM-K60
K11-only ubiquitin	Boston Biochem	Cat#UM-K110
K27-only ubiquitin	Boston Biochem	Cat#UM-K270
K29-only ubiquitin	Boston Biochem	Cat#UM-K290
K33-only ubiquitin	Boston Biochem	Cat#UM-K330
K48-only ubiquitin	Boston Biochem	Cat#UM-K480
K63-only ubiquitin	Boston Biochem	Cat#UM-K630
Histone H2A (1-22) - GK(Biotin)	AnaSpec	Cat#64639-1
Histone H3 (1-21) Biotinylated	AnaSpec	Cat#AS-61702
Histone H4 (1-23) - GGK(Biotin)	AnaSpec	Cat#AS-65097
KOD DNA polymerase	Merck Millipore	Cat#71086-4
Phusion DNA polymerase	NEB	Cat#M0530L

**Critical Commercial Assays**		

Dual-Luciferase Reporter Assay System	Promega	Cat#E1910
UbiCREST Deubiquitinase Enzyme Kit	Boston Biochem	Cat#K-400
RNeasy Mini Kit (RNA Purification)	QIAGEN	Cat#74104
iScript cDNA synthesis kit	Biorad	Cat#170-8890
SYBR Select Master Mix	Applied Biosystems	Cat#4472908

**Deposited Data**		

Raw Imaging Data	This paper	http://dx.doi.org/10.17632/j2wjdkj5cn.1

**Experimental Models: Cell Lines**		

HEK293T	ATCC	Cat#CRL-3216
HeLa	ATCC	Cat#CCL-2
HCT116	ATCC	Cat#CCL-247

**Experimental Models: Organisms/Strains**		

*D. melanogaster*: *hyd*^*K7-19*^	Prof. Jessica E. Treisman	FlyBase: FBal0144234
*D. melanogaster*: *gro*^*MB36*^	Prof. David Ish-Horowicz	FlyBase: FBal0230454
*D. melanogaster*: *axin*^*P*^	Prof. Tetsu Akiyama	FlyBase: FBal0097414
*D. melanogaster*: *UAS.SoxF*	Dr. Fernando Casares	FlyBase: FBtp0051564
*D. melanogaster*: *pygo*^*S123*^	Bloomington Drosophila Stock Center	FlyBase: FBal0146872 Bloomington: 7209
*D. melanogaster*: *UAS.Arm*^*S10*^	Bloomington Drosophila Stock Center	FlyBase: FBtp0001723 Bloomington: 4782
*D. melanogaster*: *UAS.MamDN*	Prof. Sarah Bray	FlyBase: FBtp0014588
*D. melanogaster*: *Vg-Gal4, UAS-flp; FRT82b GFP*	Bienz Laboratory	Miller et al., 2013

**Oligonucleotides**		

Primer sequences for RT-qPCR, see Table S3	This paper	N/A

**Recombinant DNA**		

Plasmid: pCMV-tag2b-Flag-UBR5	Prof. Rina Rosin-Arbesfeld	Hay-Koren et al., 2011
Plasmid: pCS2 Myc-TLE3	Prof. Ethan Lee	Hanson et al., 2012
Plasmid: pcDNA3.1 VCP-GFP	Prof. Nico Dantuma	Tresse et al., 2010
Plasmid: pcDNA3.1 HA-Δ45-β-catenin	Bienz Laboratory	Morin et al., 1997
Plasmid: pET GST-HES1	Prof. Stefano Stifani	Grbavec and Stifani, 1996
Plasmid: pCMV His-ubiquitin	Dr. Thomas Mund	Mund et al., 2015
Plasmid: pcDNA3.1 HA-PAIP2	Bienz Laboratory	N/A
Plasmid: pcDNA3.1 SSDP-GFP	Bienz Laboratory	Fiedler et al., 2015
Plasmid: pcDNA3.1 LDB1-GFP	Bienz Labaratory	Fiedler et al., 2015
Plasmid: pcDNA3.1 GFP-TCF4	Bienz Labaratory	N/A
Plasmid: pcDNA3.1 GFP-Pygo1	Bienz Labaratory	N/A
Plasmid: pSpCas9(BB)-2A-GFP (PX458)	Addgene	Ran et al., 2013; Addgene#48138
Plasmid: pSpCas9(BB)-2A-Puro (PX459)	Addgene	Ran et al., 2013; Addgene#62988
Plasmid: pTA-Luc m50 Super 8x TopFLASH	Addgene	Veeman et al., 2003; Addgene#12456
Plasmid: pRL-CMV Renilla luciferase	Promega	Cat#E2261

### Contact for Reagent and Resource Sharing

Requests for further information or reagents should be directed to the lead contact and corresponding author, Mariann Bienz (mb2@mrc-lmb.cam.ac.uk).

### Experimental model and subject details

HEK293T, HCT116 and HeLa cells were cultured in DMEM (GIBCO), supplemented with 10% fetal bovine serum (FBS) at 37°C in a humidified atmosphere with 5% CO_2_. All cells were screened for *Mycoplasma* infection.

### Method details

#### Cell-based assays

Transient transfections of all cells were performed using polyethylenimine or Lipofectamine 2000. Wnt inductions were for 6 hr, either with Wnt3A-conditioned media or 20 mM LiCl (or 20 mM NaCl as control). Where noted, 10 μM MG132, 5 μM NMS-873, 2.5 μM CB-5083 or 50 μg mL^−1^ cycloheximide was added for the same time.

For coIP assays, cells were lysed 36 hr post-transfection in lysis buffer (20 mM Tris-HCl pH 7.4, 200 mM NaCl, 10% glycerol, 5 mM NaF, 2 mM Na_3_VO_4_, 0.2% Triton X-100, protease inhibitor cocktail). Lysates were clarified by centrifugation (16,100x *g*, 10 min), and supernatants incubated with affinity gel (Flag- or HA-) or GFP-trap for 90 min at 4°C. Subsequently, immunoprecipitates were washed 4x in lysis buffer and eluted by boiling in LDS sample buffer for 10 min. coIP assays using biotinylated histone tail peptides were conducted in similar fashion, except that lysates were incubated with 1.5 μM biotinylated peptide for 45 min, prior to the addition of streptavidin dynabeads.

Ni-NTA pull-down experiments were conducted in the above fashion, except that cells were lysed in urea buffer (8 M urea, 50 mM Na_2_HPO_4_ pH 8, 300 mM NaCl, 2 mM N-ethylmaleimide (NEM), 5 mM chloroacetamide, 0.5% NP40, 25 mM imidazole, protease inhibitor cocktail) and sonicated for 2x 10 s with a Soniprep 150 plus sonicator (MSE) prior to addition of Ni-NTA agarose. Beads were washed 6x in urea buffer and ubiquitylated proteins eluted by boiling in sample buffer.

For luciferase reporter assays (SuperTOP) assays, cells were lysed 20 hr post-transfection with SuperTOP and CMV-Renilla (control) plasmids, and analyzed with the Dual-Glo Luciferase Reporter Assay kit (Promega) according to the manufacturer’s protocol. Values were normalized to Renilla luciferase, and are shown as mean ± SEM relative to unstimulated controls (set to 1 in Figures 2A, S2D, S6A, and S6B) or to stimulated WT cells (set to 100% in Figures 2C, 2D, 4F, 6D, S2C, and S7B).

For UbiCREST assays (Hospenthal et al., 2015), the UbiCREST DUB Enzyme kit was used, following the manufacturer’s protocol. Briefly, immunoprecipitates of Flag-TLE3 or HA-Ub (generated as above) were washed twice and resuspended in ‘1X DUB reaction buffer’. Deubiquitinases were added and reactions incubated for 45 min at 37°C (while rotating), and subsequently quenched by addition of LDS sample buffer.

#### Cloning

Mutagenesis of parental plasmid DNA was carried out using standard PCR-based methods, using either KOD DNA polymerase (Merck Millipore) or Phusion DNA polymerase (NEB) and verified by sequencing.

#### CRISPR/Cas9 genome editing

HEK293T or HCT116 KO cells were generated essentially as described (Ran et al., 2013), using single-guide RNA-encoding plasmid derivatives of pSpCas9(BB)-2A-GFP (PX458) (Table S1). Cells were selected for high expression of GFP by FACS 48 hr post-transfection, and individual clones expanded in 96-well plates. Clones were screened by western blot analysis and subsequently by DNA sequencing (Table S2) to confirm the presence of frameshifting indels. To ensure consistency, multiple UBR5 and XIAP KO lines were isolated and sequenced.

For transient knockdown of β-catenin, single-guide RNAs were cloned into pSpCas9(BB)-2A-Puro (PX459). Selection with puromycin was initiated 48 hr post-transfection and carried out for 96 hr. Cells were left to recover for 72 hr prior to seeding for experiment.

#### *Drosophila* strains and analysis

Double mutant *Drosophila melanogaster* strains were generated from parental strains with standard techniques, and checked by complementation.

Fly wings were dissected and mounted in 6:5 mixture of *lactic acid*:*ethanol*, and imaged with a Nikon Eclipse TE2000-E microscope.

Wing disc clones were generated with *vg.GAL4, UAS.flp; FRT82b GFP* (also used for overexpression of UAS transgenes), as previously described (Miller et al., 2013). Wing discs were dissected from late third-instar larvae, fixed in phosphate-buffered saline (PBS) containing 4% formaldehyde, 0.1% Triton X-100 for 30 min and permeabilized in PBS containing 0.1% Triton X-100 for 5x 5 min. Discs were blocked in blocking buffer (PBS supplemented with 0.5% bovine serum albumin, 0.1% Tween-20) for 1 hr and incubated with primary antibodies in blocking buffer at 4°C. Discs were washed in blocking buffer and incubated with secondary antibodies. All discs were embedded in VectaShield with DAPI mounting media, and single confocal images acquired at identical settings with a Zeiss Confocal Microscope.

#### Immunofluorescence

HeLa cells were treated with 20 mM LiCl for 6 hr, fixed on coverslips with 4% formaldehyde and permeabilized by 0.5% Triton X-100 in PBS. Cells were then blocked in 3% bovine serum albumin in PBS, and incubated with primary antibodies. Cells were washed in blocking buffer and incubated with secondary antibody. Coverslips were washed and embedded with VectaShield with DAPI mounting media. Images were acquired at identical settings with a Zeiss Confocal Microscope.

#### *In vitro* ubiquitylation assays

*In vitro* ubiquitylation assays were conducted in 20 μL format in buffer consisting of 25 mM Tris-HCl pH 7.4, 10 mM MgCl_2_, 1 mM ATP, 200 ng UBE1A, 750 ng UBE2L3, 800 ng GST-HECT_2217-2799_-wt or GST-HECT_2217-2799_-CS, and 500 ng ubiquitin (wt, methyl- or K-only mutant). Reactions were incubated for 2 hr at 30°C and quenched by the addition of LDS sample buffer. An aliquot of each reaction was resolved via SDS-PAGE and analyzed by western blotting.

#### Mass Spectrometry

For affinity purification of UBR5-associated proteins, 20x 175 cm^2^ flasks of HEK293T cells transfected with UBR5 or control baits were used for each experiment. Cells were lysed in 40 mL lysis buffer (20 mM Tris-HCl pH 7.4, 10% glycerol, 100 mM NaCl, 5 mM NaF, 2 mM Na_3_PO_4_, 0.2% Triton X-100, protease inhibitor cocktail), and sonicated 10x 10 s at 40% intensity with a Branson 250 Sonifier. Cell lysates were clarified by centrifugation (21,000x *g*, 30 min, 4°C) and incubated (while rotating) for 2 hr with Flag affinity gel at 4°C. Immunoprecipitates were washed 5x with lysis buffer, and subsequently eluted with lysis buffer supplemented with 250 μg mL^−1^ 3xFlag-Peptide. Eluates were boiled in LDS sample buffer and resolved on 4%–12% Bis-Tris SDS-polyacrylamide gels. These were stained with Imperial Protein Stain, and gel lanes cut into 1-2 mm slices for *in situ* trypsin digestion. Resulting peptides were extracted in 2% formic acid / 2% acetonitrile mix. Digests were analyzed by nano-scale capillary LC-MS/MS using an Ultimate U3000 HPLC and C18 Acclaim PepMap100 nanoViper (Thermo Scientific Dionex). LC-MS/MS data were searched against a protein database (UniProt KB) with the Mascot search engine program (Matrix Science). MS/MS data were validated using the Scaffold program (Proteome Software).

#### Protein expression and purification

GST-tagged recombinant proteins were purified from BL21 (DE3) pRIL *E. coli* bacterial strains. Bacteria were grown in LB media supplemented with appropriate antibiotic to an OD_600_ of 0.7 and induced by addition of 0.4 mM isopropyl-β-D-1thiogalactopyranoside (IPTG). Proteins were expressed for 6 hr at 37°C or for 12 hr at 22°C. Cells were resuspended in lysis buffer (25 mM Tris-HCl pH 8, 200 mM NaCl, 10% glycerol, 5 mM β-mercaptoethanol, 10 μg mL^-1^ DNase, protease inhibitor cocktail) and lysed by high-pressure homogenization with an Emulsiflex C-3. Lysates were clarified by ultracentrifugation (140,000x *g*, 30 min, 4°C) and mixed with glutathione Sepharose 4B. Beads were washed 7x with lysis buffer, including a high salt (500 mM NaCl) fourth wash, and GST-tagged protein eluted with 20 mM L-glutathione (reduced). All proteins were purified by size exclusion chromatography, and purity was assessed by SDS-PAGE prior to use.

#### RT-qPCR

HEK293T cells were treated with 20 mM LiCl (or 20 mM NaCl) for 6 hr. RNA was extracted with the RNeasy mini kit and converted to cDNA using the iScript cDNA synthesis kit, as described in the manufacturers protocol. RT-qPCR reactions were run in 20 μL, 96-well format on a Vii7 Real-Time PCR System (Applied Biosystems) using SYBR Select Mix with the primer pairs listed (Table S3). Values were normalized to *PMM1* (Hanson et al., 2012), and are shown as mean ± SEM relative to unstimulated controls (set to 1).

### Quantitation and Statistical Analysis

All error bars are represented as mean ± SEM for at least three independent experiments. Statistical significance was calculated by Student’s t test and denoted as follows: ^∗^ = p < 0.01, ^∗∗^ = p < 0.001.

### Data and Software Availability

Original uncropped imaging data have been deposited to Mendeley Data and are available at http://dx.doi.org/10.17632/j2wjdkj5cn.1.

## Author Contributions

J.E.F. and N.N. performed the biochemical analysis and the cell-based assays, J.M. conducted the genetic analysis in *Drosophila*, and M.B. conceived and supervised the study and wrote the manuscript with input from all authors.

## Supplemental Information

Document S1. Figures S1–S7 and Tables S1–S3

Document S2. Article plus Supplemental Information
