# Supplementary material for: Wnt-Dependent Inactivation of the Groucho/TLE Co-repressor by the HECT E3 Ubiquitin Ligase Hyd/UBR5
Source: Mol Cell. 2017 Jul 20;67(2):181–193.e5. doi: 10.1016/j.molcel.2017.06.009 (PMC5592244; doi:10.1016/j.molcel.2017.06.009)
Supplement: Document S1. Figures S1–S7 and Tables S1–S3 [file mmc1.pdf]

Molecular Cell, Volume *67*

Supplemental Information

Wnt-Dependent Inactivation of the Groucho/TLE Co-repressor by the HECT E3 Ubiquitin Ligase Hyd/UBR5

Joshua E. Flack, Juliusz Mieszczanek, Nikola Novcic, and Mariann Bienz

SUPPLEMENTAL INFORMATION

Fig. S1 (related to main **Figs. 1 & 3**)

Additional clonal analysis in wing discs. (**A-C**) Basal views of wing discs bearing (**A, B**) *hyd*$^{K7\text{-}19}$ mutant clones (marked by absence of GFP, *green*), (**B**) also overexpressing SoxF, or (**C**) *hyd*$^{K7\text{-}19}$ *gro*^{MB36} double-mutant clones, co-stained with DAPI (*blue*) and antibodies as indicated in panels (in color, as in merges). The *hyd*-dependent overgrowths are particularly pronounced in the prospective hinge region (*large red arrows* in **A**), but are also evident in *hyd* clones within the prospective blade and margin zones (*small red arrow* in **A**); they tend to be accompanied by Wg derepression (*red arrows* in **A**), however they do not depend on this derepression since clones in the proximal hinge zone (*white arrowheads* in **A**) show overgrowth without Wg derepression. These overgrowths (especially those in the distal hinge zone, adjacent to the wing blade) are partially suppressed by SoxF (*white arrows* in **B**) whereby the degree of suppression depends on the level of SoxF expression, or completely suppressed by Groucho loss (*white arrows* in **C**). Recall that Wg is initially expressed throughout the prospective wing blade and within the adjacent zone of the hinge region (i.e. its distal zone) in early larval discs, but undergoes negative autoregulation to narrow its own expression progressively within the prospective hinge and margin zones to thin stripes (see main text and **Fig. 1B**); the Wg stripes in the margin are outside the focal planes of the images in (**B, C**) and are thus barely visible (as these images were focused on clones near the hinge regions that exhibit suppressed overgrowth). Size bars, 50 μm. (**D-F**) Sections of wing discs from late third instar larvae, fixed and co-stained with DAPI (*blue*) and antibodies as indicated above panels (in color, as in merges), bearing (**D**) *hyd*$^{K7\text{-}19}$ *axin*^{P} double-mutant clones, lacking Sens (*asterisk* in **D**) and showing derepressed Wg (*arrow* in **D**), similarly to *hyd*$^{K7\text{-}19}$ single-mutant clones ± overexpressed Arm^{S10} (see **Fig. 1D, F**); (**E**) *hyd*$^{K7\text{-}19}$ mutant clones, showing normal Armadillo expression levels; or (**F**) *hyd*$^{K7\text{-}19}$ mutant clones also expressing dominant-negative Mastermind (dnMam) to block Notch-dependent transcription (Helms et al., 1999); the efficacy of this blockade is evident in wt territories near the margin (*arrowheads* in **F**) where expression of Wg (and Sens as a consequence) is abolished. However, the dnMam-expressing *hyd* clones retain derepression of Wg (*arrows* in **F**), arguing against this derepression being a consequence of hyperactive Notch signaling. Size bars, 10 μm.

Fig. S2 (related to main **Fig. 2**)

Analysis of HEK293T KO cells lacking different HECT E3 ligases. (**A**) Cartoons of HECT E3 ligases previously linked to Wnt signaling (HUWE1, HECTD1, UBE3C; de Groot et al., 2014; Tran et al., 2013; Wen et al., 2015) or UBR5 (TRIP12; Gudjonsson et al., 2012), with domains indicated (residue numbers of human proteins shown underneath). (**B**) Western blots of lysates from KO lines (see also **Tables S1 & S2**, and STAR Methods), probed with antibodies as indicated, to demonstrate absence of gene product; the following antibodies were used: α-HUWE1, α-TRIP12, α-HECTD1, α-UBE3C (Abcam). (**C, D**) SuperTOP assays of (**C**) two independently isolated UBR5 KO lines compared to parental control (*above*, corresponding Western blot), stimulated with Wnt3A or LiCl for 6 hours (mean relative luciferase values of induced control cells were set to 100%), or (**D**) wt or UBR5 KO cells expressing wt or catalytically-dead (CS) GFP-UBR5 (*below*, corresponding Western blot); fold induction indicates values relative uninduced controls; error bars, SEM of >3 independent experiments; ** = $p < 0.001$, * = $p < 0.01$.

Fig. S3 (related to main **Fig. 3**)

Identification of TLE3 as a UBR5-interacting protein. (**A**) SDS-PAGE gel stained with Coomassie Blue, showing proteins coIPed with catalytically-dead FLAG-UBR5 (UBR5-CS) ± MLLE or control baits (after expression in HEK293T cells stimulated with LiCl for 6 hours). (**B**) Selected significant UBR5-specific hits identified in two independent experiments by LC-MS/MS mass spectrometry, essentially as described (Fiedler et al., 2015), but see also STAR Methods, for further details; shown are unweighted spectral counts >95% probability; only hits with PAM2 motifs previously identified as putative MLLE-binding proteins are listed, in comparison to TLE3 (the only hit linked to Wnt/β-catenin signaling). (**C**) CoIP assay as in main **Fig. 4B**, confirming association between TLE3 and UBR5, as found in (**B**). (**D**) Assay as in main **Fig. 3A**, showing LiCl-dependent ubiquitylation of endogenous TLE3. (**E, F**) CoIP assays as in main **Fig. 4B**, showing constitutive association between FLAG-UBR5-CS and (**E**) GFP-TCF4, or (**F**) GFP-PYGO1.

Fig. S4 (related to main **Fig. 5**)

K48-Ub specificity of UBR5-HECT, and TLE3 stability. (**A**) *In vitro* Ub assay with bacterially expressed GST-UBR5$_{2217\text{-}2799}$ or catalytically-inactive GST-UBR5-CS$_{2217\text{-}2799}$, incubated for 2 hours with wt or methylated Ub, or with K-only Ub mutants (as indicated above), and supplemented with E1 and E2 (UBE2L3) enzymes, essentially as described (Mund and Pelham, 2009); shown is a Western blot probed with α-GST, revealing Ub-GST-HECT. Note that the autoubiquitylation

achieved with K48-only Ub recapitulates the full activity of wt Ub, while all other K-only mutants only produce multi-monoubiquitylated GST-HECT (as obtained with methyl-Ub), indicating that UBR5-HECT generates exclusively K48-linked Ub chains. Note also that a shorter construct (GST-UBR5$_{2390-2799}$) was completely inactive in this assay, and GST-UBR5$_{2217-2799}$-ΔMLLE showed only weak activity (N. N., unpublished results). (**B, C**) Cycloheximide (CHX) chase experiments; shown are Western blots of lysates from (**B**) UBR5 KO cells or parental controls, or (**C**) UBR5 KO cells overexpressing wt or GFP-UBR5-CS, lysed 0-10 hours after treatment with 50 μg ml^{-1} cycloheximide plus 20 mM LiCl (or NaCl as control), probed with antibodies as indicated.

Fig. S5 (related to main **Fig. 5**)

Ligand associations of ubiquitylated K-only mutant WD40 domains. (**A**) Sequences of WD40 domains from human TLE1-4, with conserved K residues highlighted in red. (**B**) *In vivo* Ub assay in LiCl-treated UBR5 KO cells co-expressing wt or K-only HA-WD40 mutants with His-Ub, ± FLAG-UBR5 as indicated; note the varying levels of ubiquitylation of the K-only mutants (which share 14 of 16 K>R substitutions; KallR, all 16 K residues were substituted with R). (**C**) Heat-maps of the WD40 domain (2CE9; Jennings et al., 2006; *top*, ligand-binding surface; *bottom*, back side), indicating Ub levels of K-only mutants (shown in **B**); note that K720 engages in direct contacts with ligands (see main text). (**D, E**) CoIP assays as in main **Fig. 4B**, after co-expression of K-only HA-WD40 mutants with His-Ub and GFP-tagged (**D**) HES1 or (**E**) LDB1 and SSDP, as indicated, showing comparable associations of these ligands with HA-WD40, regardless of its ubiquitylation. (**F**) Pull-down assays with biotinylated histone tails (as indicated above panels; see also Chodaparambil et al., 2014), showing comparable associations of these tails with ubiquitylated and unmodified HA-TLE3.

Fig. S6 (related to main **Fig. 6**)

Additional analysis of Wnt responses after VCP/p97 inhibition. (**A, B**) SuperTOP assays as in main **Fig. 2A**, showing that (**A**) the activity of UBR5 in restoring efficient β-catenin-dependent transcription in UBR5 KO cells is highly sensitive to VCP/p97 inhibition and (**B**) dominant-negative VCP/p97 attenuates β-catenin-dependent transcription in HEK293T cells; error bars, SEM; * = $p<0.01$, ** = $p<0.001$ (in all panels). (**C**) RT-qPCR assays as in main **Fig. 2B**, showing that the inducibility of endogenous Wnt target genes is attenuated (*AXIN2*, *SP5*) or blocked (*NKD1*) by VCP/p97 inhibition in HEK293T cells, while this treatment barely effects the residual induction of these genes in UBR5 KO cells (except for *AXIN2* which retains NMS-873 sensitivity in these cells,

but *AXIN2* also retains moderate inducibility in UBR5 KO cells, consistent with the notion that UBR5 may be redundant with another E3 ligase that ubiquitylates Groucho/TLE to target it for removal from the Wnt enhanceosome by VCP/p97). We also attempted to delete VCP/p97 in HEK293T cells by CRISPR/Cas9, to examine Wnt responses in VCP/p97 null mutant cells, but did not succeed in isolating any KO cell lines as VCP/p97 KO appeared to be cell-lethal (J. E. F., unpublished data).

Fig. S7 (related to main **Fig. 3**)

TLE3 ubiquitylation and Wnt responses in XIAP KO cells. (**A**) TLE3-Ub assays in XIAP KO cells as in main **Fig. 3A**, showing that the UBR5-dependent ubiquitylation of TLE3 is independent of XIAP. (**B**) SuperTOP assays as in main **Fig. 2A**, showing that UBR5 KO reduces the Wnt- and LiCl-inducibility of HEK293T more severely than XIAP KO; error bars, SEM; ** = $p<0.001$.

Table S1 (related to main **Fig. 2**)

gRNA sequences used for CRISPR/Cas9-mediated genome engineering

Table S2 (related to main **Fig. 2**)

Genotyping primer pairs used for CRISPR/Cas9-mediated genome engineering

Table S3 (related to main **Fig. 2**)

Primer pairs used for RT-qPCR

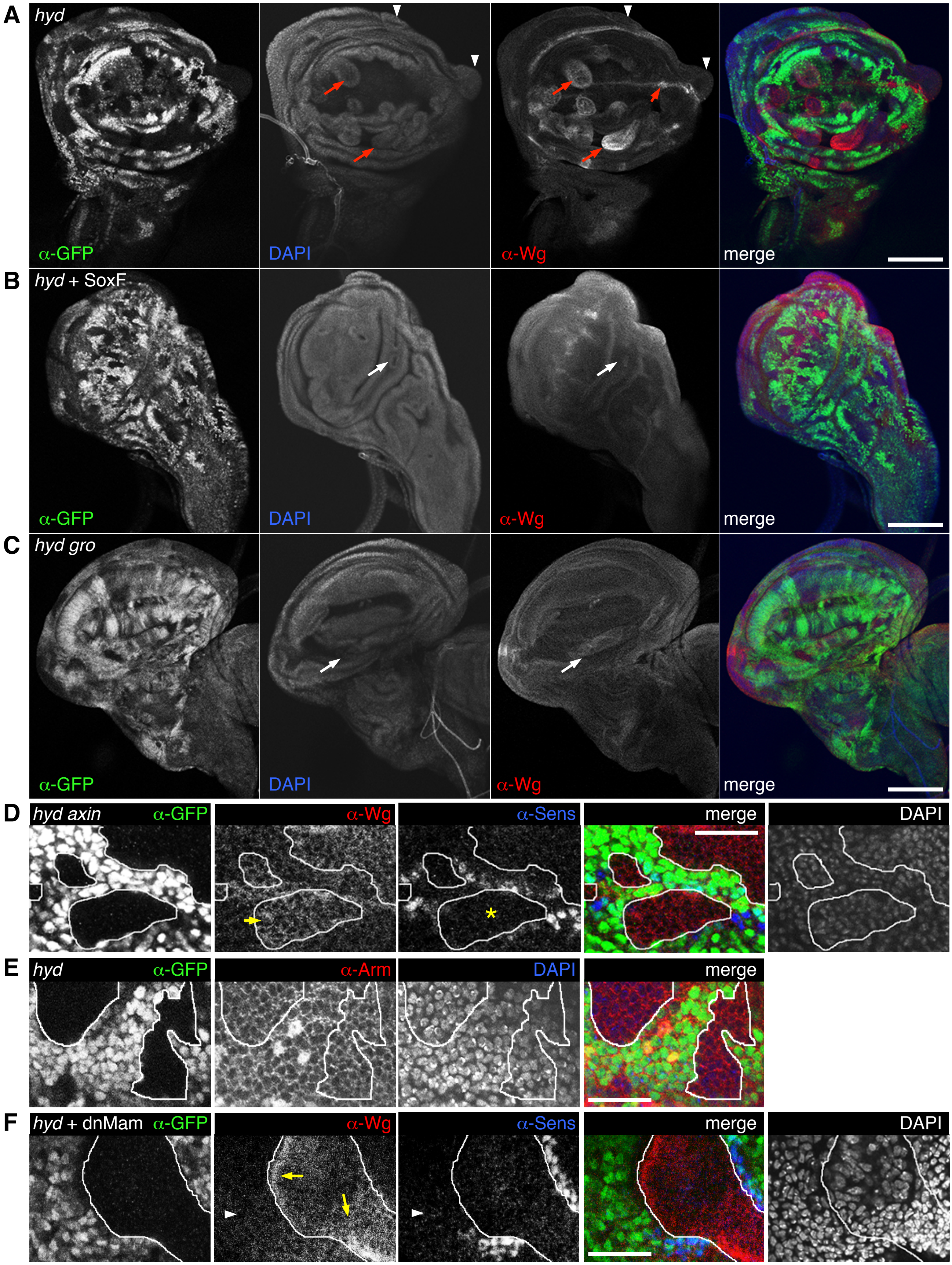
A
hyd
α-GFP
DAPI
α-Wg
merge
B
hyd + SoxF
α-GFP
DAPI
α-Wg
merge
C
hyd gro
α-GFP
DAPI
α-Wg
merge
D
hyd axin
α-GFP
α-Wg
α-Sens
merge
DAPI
E
hyd
α-GFP
α-Arm
DAPI
merge
F
hyd + dnMam
α-GFP
α-Wg
α-Sens
merge
DAPI

Figure S1

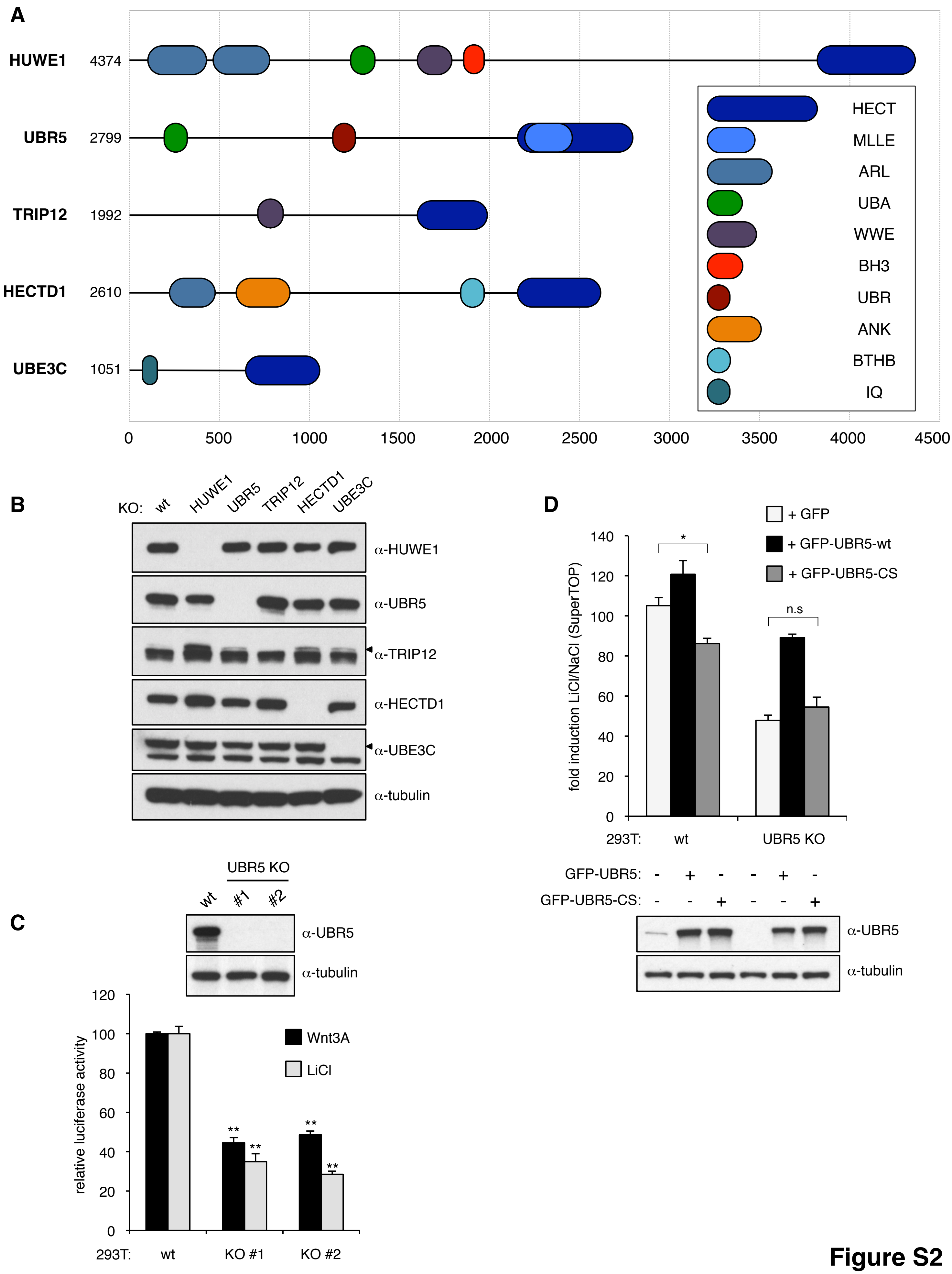
A
HUWE1 4374
UBR5 2799
TRIP12 1992
HECTD1 2610
UBE3C 1051
HECT
MLLE
ARL
UBA
WWE
BH3
UBR
ANK
BTHB
IQ
0
500
1000
1500
2000
2500
3000
3500
4000
4500
B
KO: wt HUWE1 UBR5 TRIP12 HECTD1 UBE3C
α-HUWE1
α-UBR5
α-TRIP12
α-HECTD1
α-UBE3C
α-tubulin
C
UBR5 KO
wt #1 #2
α-UBR5
α-tubulin
relative luciferase activity
120
100
80
60
40
20
0
Wnt3A
LiCl
**
**
**
**
293T: wt KO #1 KO #2
D
+ GFP
+ GFP-UBR5-wt
+ GFP-UBR5-CS
fold induction LiCl/NaCl (SuperTOP)
140
120
100
80
60
40
20
0
*
n.s
293T: wt UBR5 KO
GFP-UBR5: - + - - + -
GFP-UBR5-CS: - - + - - +
α-UBR5
α-tubulin

Figure S2

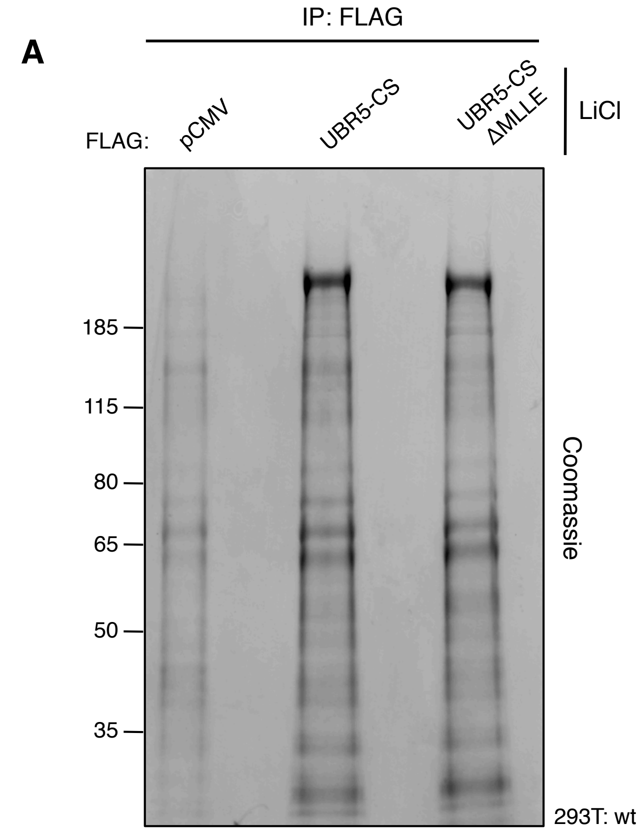

B

Identified Protein	pCMV	UBR5-CS	UBR5-CS ΔMLLE
UBR5	11	1961	1160
BUB1β	-	23	7
RANGAP1	-	11	12
BUB1	-	11	2
p53	-	6	5
RANBP1	-	6	3
WDR48	-	5	-
CEP78	-	3	2
ATX2L	-	2	-
TLE3	-	2	2

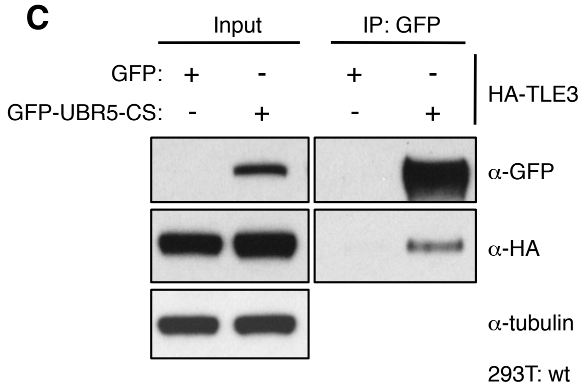

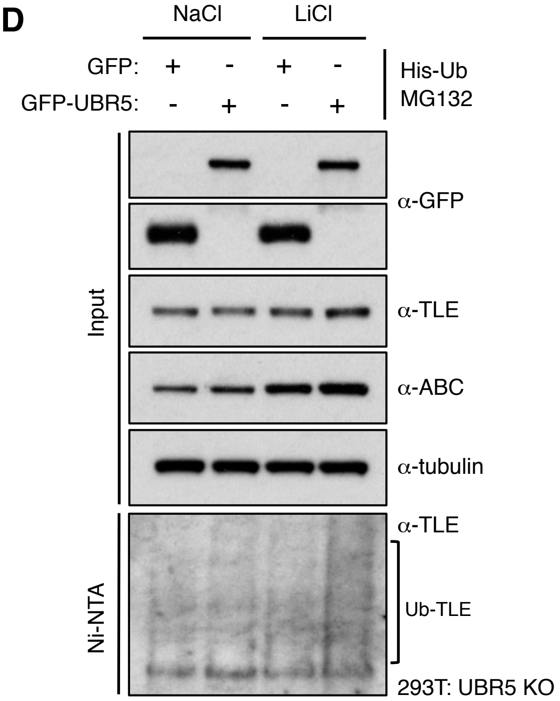

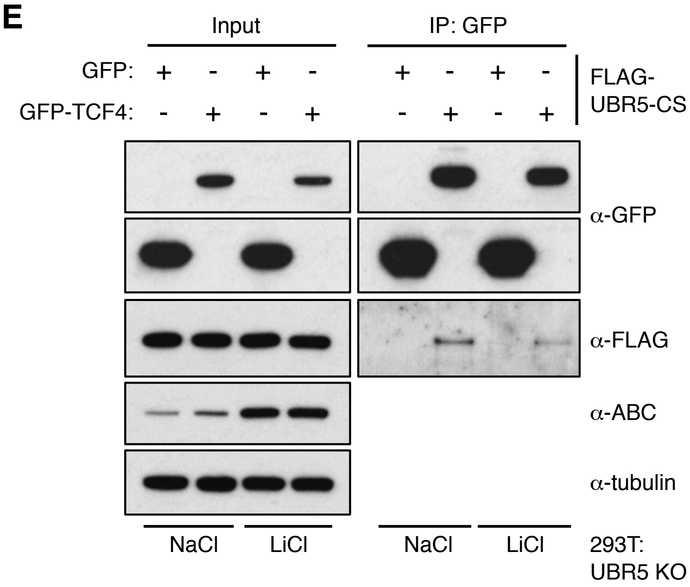

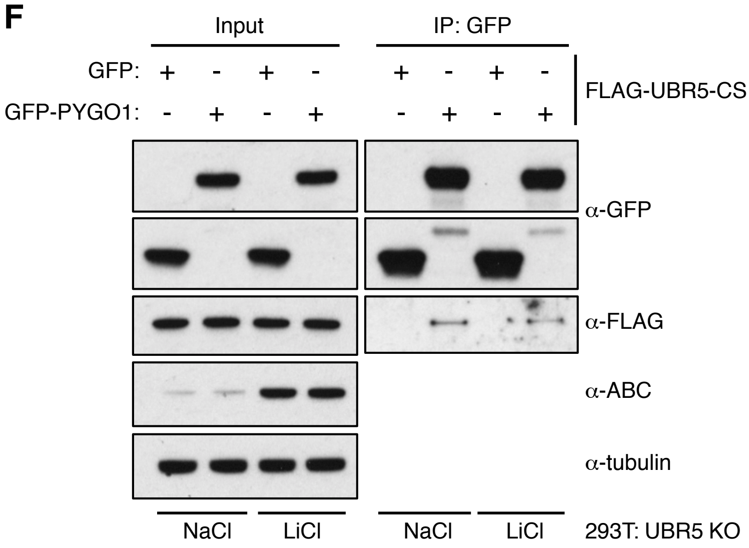

Figure S3

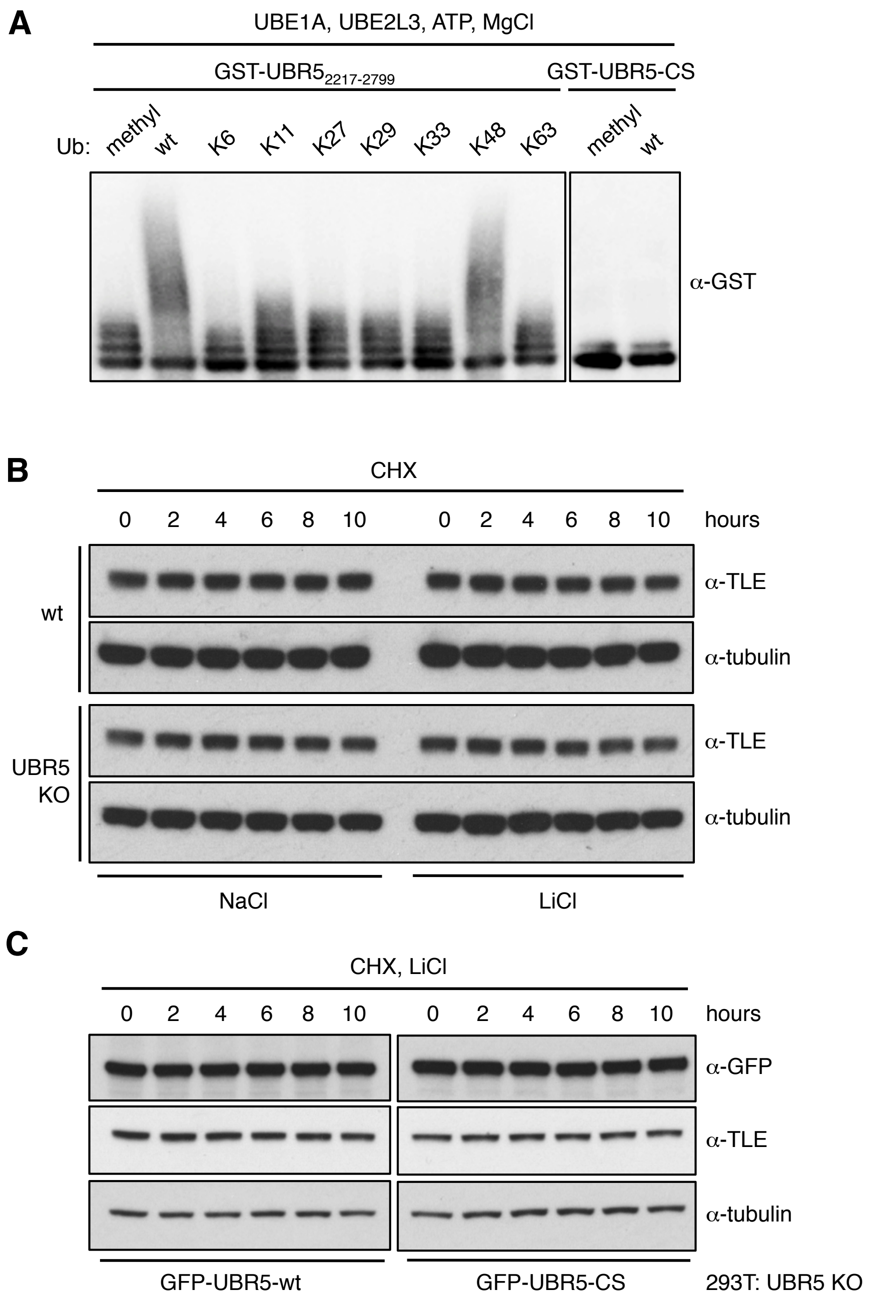
A
UBE1A, UBE2L3, ATP, MgCl
GST-UBR5 2217-2799
GST-UBR5-CS
Ub:
methyl
wt
K6
K11
K27
K29
K33
K48
K63
methyl
wt
α-GST
B
CHX
0 2 4 6 8 10 0 2 4 6 8 10 hours
wt
UBR5 KO
α-TLE
α-tubulin
α-TLE
α-tubulin
NaCl
LiCl
C
CHX, LiCl
0 2 4 6 8 10 0 2 4 6 8 10 hours
α-GFP
α-TLE
α-tubulin
GFP-UBR5-wt
GFP-UBR5-CS
293T: UBR5 KO

Figure S4

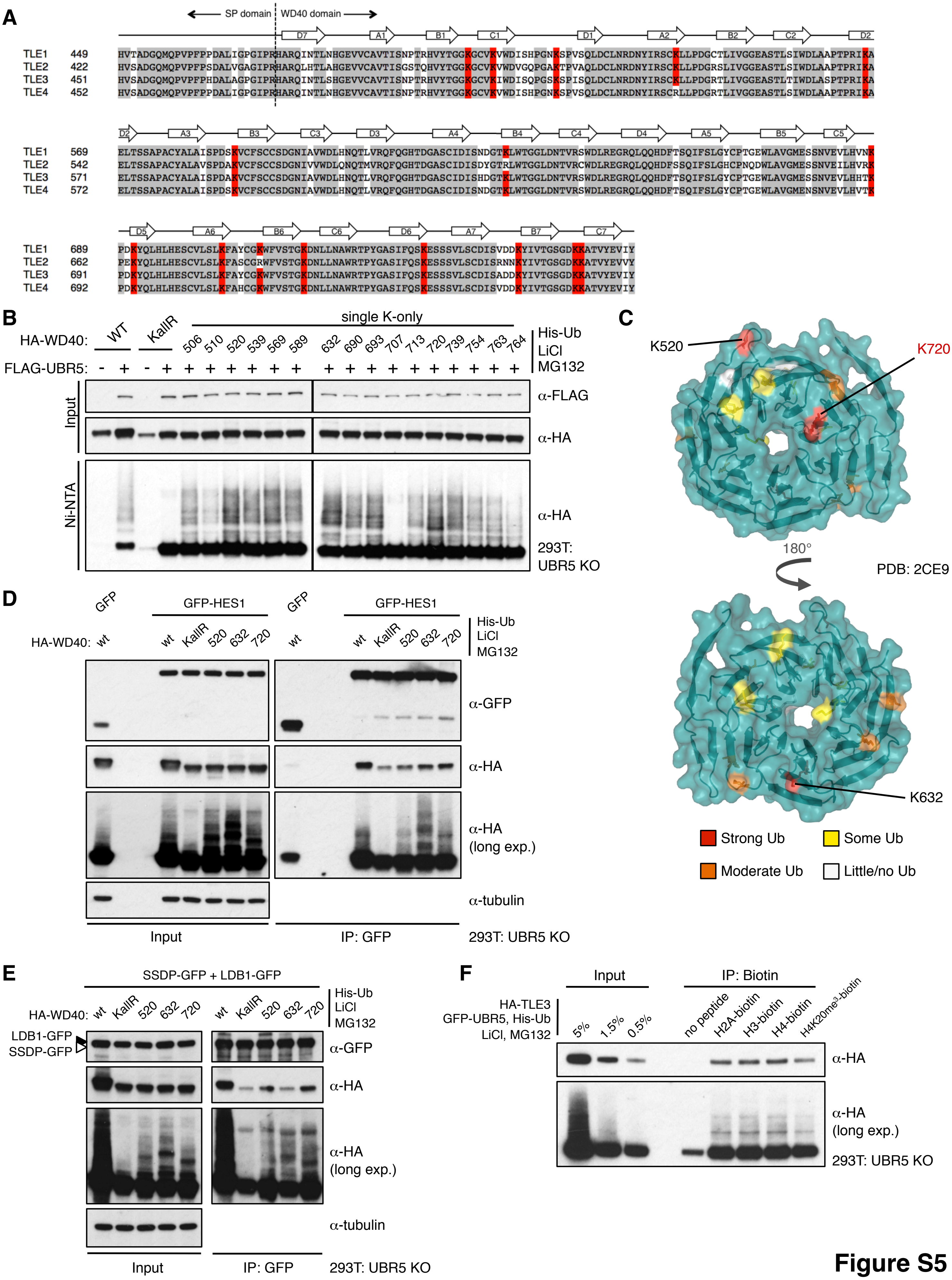

A
SP domain
WD40 domain
TLE1
TLE2
TLE3
TLE4
B
HA-WD40:
WT
KallR
single K-only
FLAG-UBR5:
His-Ub
LiCl
MG132
Input
Ni-NTA
α-FLAG
α-HA
293T: UBR5 KO
C
K520
K720
K632
180°
PDB: 2CE9
Strong Ub
Some Ub
Moderate Ub
Little/no Ub
D
GFP
GFP-HES1
HA-WD40:
α-GFP
α-HA
α-HA (long exp.)
α-tubulin
Input
IP: GFP
293T: UBR5 KO
E
SSDP-GFP + LDB1-GFP
LDB1-GFP
SSDP-GFP
F
Input
IP: Biotin
HA-TLE3
GFP-UBR5, His-Ub
LiCl, MG132
5%
1.5%
0.5%
no peptide
H2A-biotin
H3-biotin
H4-biotin
H4K20me3-biotin

Figure S5

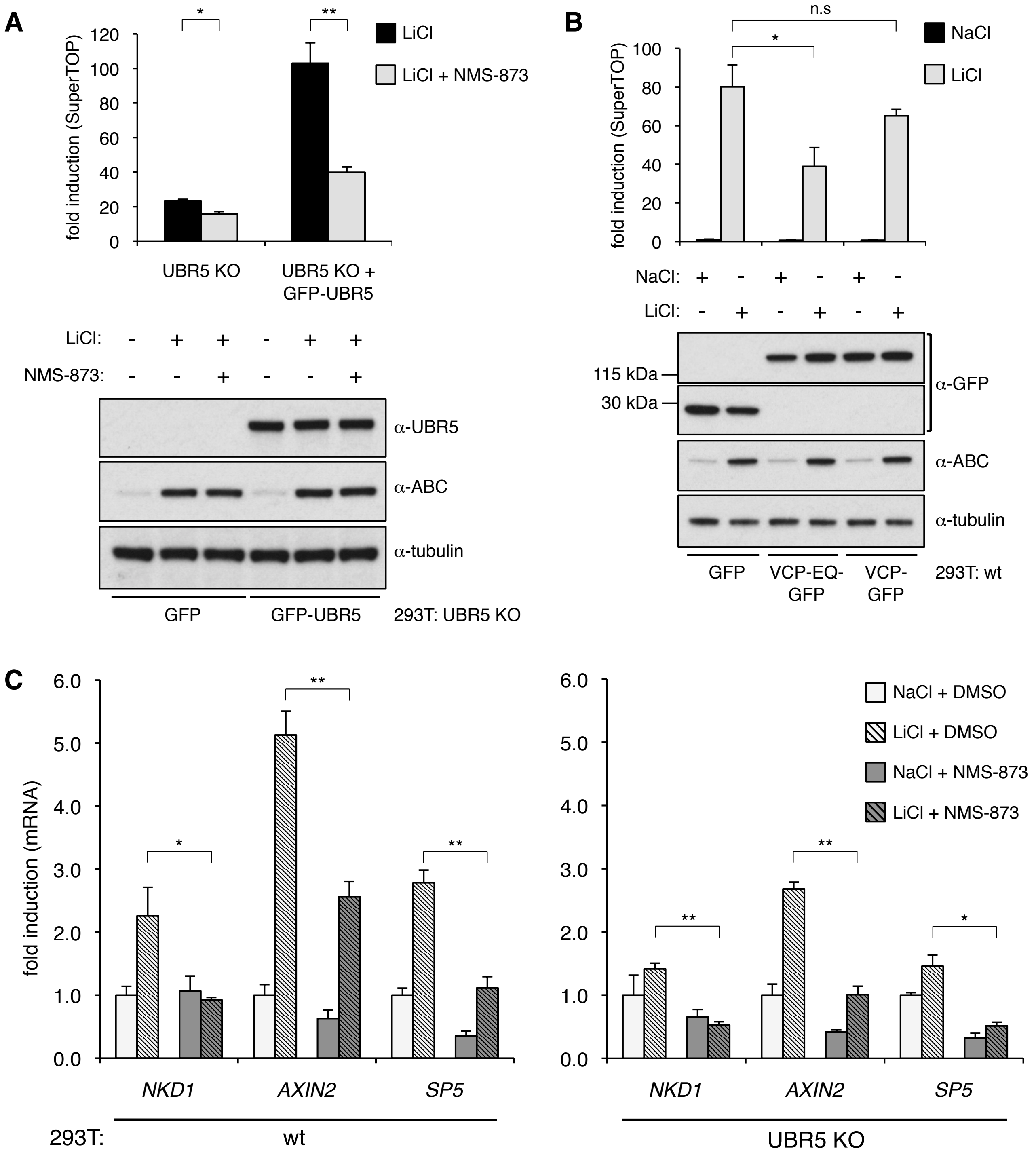
A
fold induction (SuperTOP)
120
100
80
60
40
20
0
*
**
LiCl
LiCl + NMS-873
UBR5 KO
UBR5 KO + GFP-UBR5
LiCl: - + + - + +
NMS-873: - - + - - +
α-UBR5
α-ABC
α-tubulin
GFP
GFP-UBR5
293T: UBR5 KO
B
n.s
*
NaCl
LiCl
fold induction (SuperTOP)
100
80
60
40
20
0
NaCl: + - + - + -
LiCl: - + - + - +
115 kDa
30 kDa
α-GFP
α-ABC
α-tubulin
GFP
VCP-EQ-GFP
VCP-GFP
293T: wt
C
fold induction (mRNA)
6.0
5.0
4.0
3.0
2.0
1.0
0.0
*
**
**
NKD1
AXIN2
SP5
293T:
wt
NaCl + DMSO
LiCl + DMSO
NaCl + NMS-873
LiCl + NMS-873
**
**
*
UBR5 KO

Figure S6

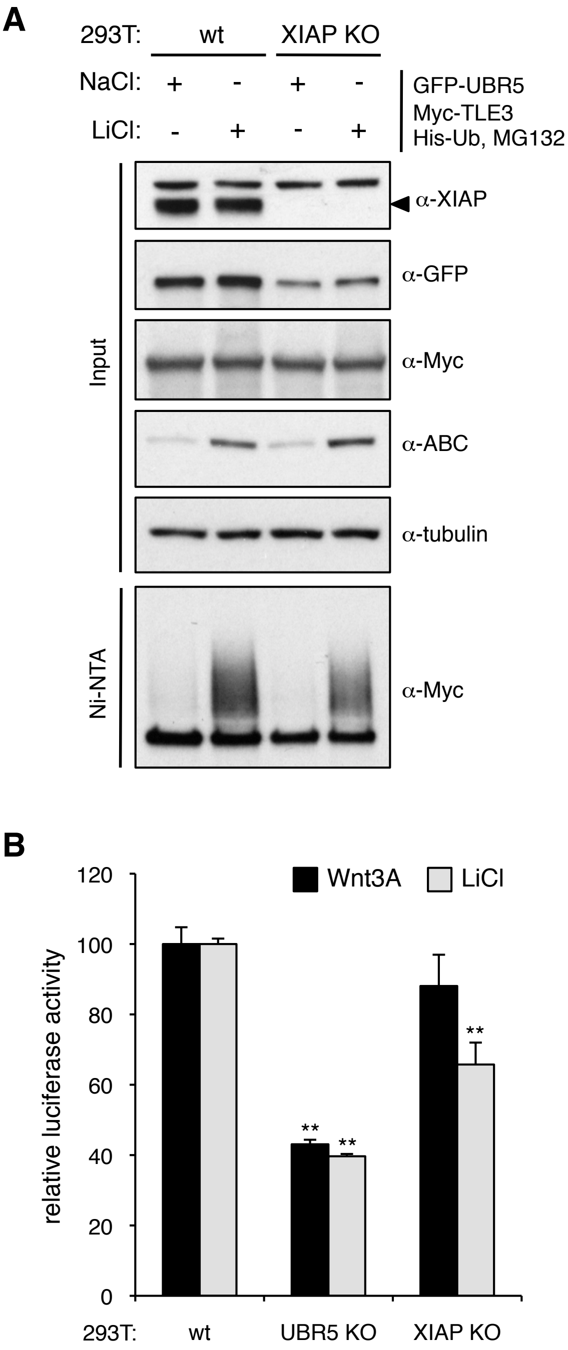
A
293T: wt XIAP KO
NaCl: + - + -
LiCl: - + - +
GFP-UBR5
Myc-TLE3
His-Ub, MG132
Input
α-XIAP
α-GFP
α-Myc
α-ABC
α-tubulin
Ni-NTA
α-Myc
B
Wnt3A
LiCl
relative luciferase activity
120
100
80
60
40
20
0
**
**
**
293T: wt UBR5 KO XIAP KO

Figure S7

Cell line	Gene	Exon Targeted	gRNA sequence
HEK293T	*UBR5*	6	(G)CTGGAGCTCGAGATTCCCGC
HEK293T	*HUWE1*	5	GGACCGCTTCGATGGAATAC
HEK293T	*TRIP12*	3	(G)CTGACTCCGTGAACCGCCAG
HEK293T	*HECTD1*	3	(G)TATCTGCGGAATGTACCCGA
HEK293T	*UBE3C*	4	GCTACCTTGTCACAGTCCGG
HEK293T	*XIAP*	2	(G)TATCAGACACCATATACCCG
HEK293T	*CTNNB1*	3	GAAAAGCGGCTGTTAGTCAC
HCT116	*UBR5*	6	(G)CTGGAGCTCGAGATTCCCGC

Table S1

Gene	Exon	Primer sequence	
UBR5	6	Forward	GATTGAGCCCGGGAGTTTTG
		Reverse	TCCATCTTCATCATCCCGGC
		Seq	TGAGGCAGGAGGATCACTTC
HUWE1	5	Forward	GCAGATCAAAACATGGAACATTGG
		Reverse	CTCTATGGAACTGTACAGATGCCG
		Seq	GTATGACAATGAACTACAGC
TRIP12	3	Forward	AAGCTGCAGTTCATCATCTGCT
		Reverse	TTGCTAATTTGGCCTGTAATCCAGAA
		Seq	GCGCAGTGCTAGTCCAGACT
HECTD1	3	Forward	GACTACAGGTGCCTGTCACC
		Reverse	ACTACCAGGAACTGAAGTGCAC
		Seq	ATATGATTTCTTTCACTACAG
UBE3C	4	Forward	GAAGAAAGGCGAAGGTTGAAAAATGC
		Reverse	CACATCACCACATAGGTAACCTCTC
		Seq	CTACAATTCAACTGTGAGCA
XIAP	2	Forward	AAACTTGTGTACCTGCAGACA
		Reverse	CCGTGCTTCATAATCTGCCA
		Seq	CTTTTGCTAATTTTCCAAGTGG

Table S2

Gene	Primer sequence	
PMM1	Forward	CTCCTAGTGGCACTGGCTTC
	Reverse	GCAGGCTAGATCTCGTACCG
NKD1	Forward	GCTGAGCGTGTCTCTCAACA
	Reverse	AGGAGTGGATCGGGAGACAG
AXIN2	Forward	CTGGTGCAAAGACATAGCCA
	Reverse	GTCCAGCAAAACTCTGAGGG
SP5	Forward	TCGGACATAGGGACCCAGTT
	Reverse	CTGACGGTGGGAACGGTTTA

Table S3
